# Van der Waals Template‐Assisted Growth of Two‐dimensional Sb_2_S_3_

**DOI:** 10.1002/advs.202509903

**Published:** 2025-09-29

**Authors:** Sindhu Priya Giridhar, Irfan H. Abidi, Jiawen Qiu, Ghalib Alfaza, Jonathan O. Tollerud, Pargam Vashishtha, Jianfeng Mao, Edwin LH Mayes, Billy J. Murdoch, Mei Xian Low, Yuxiao Hou, Taimur Ahmed, Jeffrey A. Davis, Enrico Della Gaspera, Priyank Kumar, Lu‐Tao Weng, Sumeet Walia

**Affiliations:** ^1^ Centre for Opto‐electronic Materials and Sensors (COMAS) School of Engineering RMIT University 124 La Trobe Street Melbourne Victoria 3001 Australia; ^2^ Materials Characterization and Preparation Facility (GZ) The Hong Kong University of Science and Technology (Guangzhou) Guangzhou Guangdong 511400 China; ^3^ Particles and Catalysis Research Group School of Chemical Engineering, The University of New South Wales Sydney NSW 2052 Australia; ^4^ Optical Sciences Centre Swinburne University of Technology Victoria 3122 Australia; ^5^ Department of Electrical Engineering and Computer Science University of Arkansas Fayetteville AR 72701 USA; ^6^ RMIT Microscopy and Microanalysis Facility RMIT University Melbourne 3000 Australia; ^7^ School of Science RMIT University 124 La Trobe Street Melbourne 3000 Australia; ^8^ Thrust of Advanced Materials Function Hub, Guangzhou Municipal Key Laboratory of Materials Informatics The Hong Kong University of Science and Technology (Guangzhou) Guangzhou Guangdong 511400 China

**Keywords:** 2D materials, CVD, MoS_2_, Sb_2_S_3_, template growth, van der Waals

## Abstract

Antimony sulfide (Sb_2_S_3_), a representative of the pnictogen chalcogenide family, possesses a tunable bandgap, strong optical absorption, and phase‐change functionality, making it a promising candidate for next‐generation optoelectronic and memory devices. However, its intrinsic quasi‐one‐dimensional (1D) crystal structure favors nanowire or nanorod growth, hindering synthesis in two‐dimensional (2D) form and limiting integration into ultrathin planar device architectures. Here, van der Waals (vdW) template‐assisted growth of atomically thin 2D Sb_2_S_3_ nanosheets on monolayer molybdenum disulfide (MoS_2_) single crystals is demonstrated, using a low‐temperature chemical vapor deposition process. The density functional theory calculations reveal that MoS_2_ lowers diffusion barriers and weakens precursor molecules adsorption, promoting lateral diffusion and 2D growth while suppressing thermodynamically favored 1D morphologies. The resulting 2D Sb_2_S_3_ exhibits sub‐8 nm thickness and with lateral dimensions dictated by the underlying MoS_2_ single‐crystal template. Remarkably, devices fabricated on the resulted Sb_2_S_3_ integrated MoS_2_ heterostructure demonstrate broadband photodetection from ultraviolet to near‐infrared, with photoresponsivity enhanced by two orders of magnitude and improved field‐effect mobility compared to bare monolayer MoS_2_. These results establish a scalable route to access 2D forms of quasi‐1D chalcogenides, bridging the critical gap between theoretical predictions and practical applications while enabling their integration into ultrathin, interface‐engineered optoelectronic and memory devices.

## Graphical abstract

Van der Waals (vdW) template‐assisted growth of atomically thin two‐dimensional (2D) Sb_2_S_3_ on monolayer MoS_2_ single crystals is demonstrated, overcoming its thermodynamically preferred one‐dimensional (1D) growth. The resulting sub‐8 nm nanosheets show uniform coverage, atomically sharp interfaces, and enhanced broadband optoelectronic performance as Sb_2_S_3_/MoS_2_ heterostructures, offering a scalable route to 2D pnictogen chalcogenides.

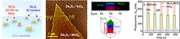

## 1 Introduction

Atomically thin two‐dimensional (2D) materials have gained immense attention due to their unique physical and chemical properties, which emerge from their reduced dimensionality and strong quantum confinement effects.^[^ ^1^, ^2^, ^3^, ^4^, ^5^ ^]^ These materials exhibit layer‐dependent electronic band structures, high carrier mobility, and strong light‐matter interactions, making them highly promising for applications in nanoelectronics, optoelectronics, energy storage, quantum, and photonic devices.^[^ ^6^, ^7^, ^8^, ^9^, ^10^, ^11^, ^12^ ^]^ While transition metal dichalcogenides (TMDs) are widely studied for their tunable electronic and optical properties,^[^ ^13^, ^14^, ^15^, ^16^ ^]^ pnictogen chalcogenides (M_2_X_3_, where M = group V metal and X = group VI chalcogen), are an emerging yet relatively underexplored class of 2D materials. Owing to their intrinsically anisotropic crystal structures and broad tunability in optoelectronic behavior, pnictogen chalcogenides present unique opportunities for functional diversification and scalable integration in next‐generation electronic and photonic devices.^[^ ^17^, ^18^, ^19^, ^20^, ^21^ ^]^ Among them, antimony sulfide (Sb_2_S_3_) has gained increasing interest due to its tunable bandgap (≈1.7–2.8 eV), high absorption coefficient (1.8 × 10^5^ cm^−1^ at 450 nm), stability in ambient conditions, and its potential for use in photodetectors, photovoltaics, and phase‐change memory applications.^[^ ^22^, ^23^, ^24^, ^25^, ^26^ ^]^ However, despite these incredible properties, its intrinsic quasi‐one‐dimensional (1D)m crystal structure makes it extremely challenging to grow as a layered 2D material. Sb_2_S_3_ consists of ribbon‐like chains of [Sb_4_S_6_]_n_ aligned along the [010] crystallographic direction, which are covalently bonded along one axis but weakly held together by van der Waals (vdW) interactions, leading to its preferential growth along the z‐axis into 1D nanostructures such as nanowires, ribbons, or thin films from three‐dimensional (3D) particulates, instead of forming continuous 2D nanosheets in lateral configuration.^[^ ^27^, ^28^, ^29^, ^30^, ^31^ ^]^ Beyond overcoming this synthesis challenge, realizing Sb_2_S_3_ in a 2D form offers superior compatibility with planar device architectures, wafer‐scale fabrication, and seamless integration with other 2D materials for complementary metal–oxide–semiconductor (CMOS) technologies. Confining the quasi‐1D chains within an ultrathin geometry enhances in‐plane anisotropic responses, tunes interchain coupling, and enables atomically sharp vdW heterostructures with tailored charge‐transfer properties critical for next‐generation atomically thin, interface‐driven devices.

Although liquid‐phase exfoliation, a top‐down approach, has been explored for obtaining Sb_2_S_3_ 2D nanosheets or nanoplatelets, it suffers from limited thickness control, solvent complexity, and restricted scalability, particularly in terms of lateral flake size.^[^ ^32^, ^33^, ^34^, ^35^ ^]^ Similarly, bottom‐up techniques such as chemical vapor deposition (CVD) have been employed for Sb_2_S_3_ synthesis, however, these methods predominantly favor 1D growth, yielding nanowires and nanotubes, rather than lateral 2D sheets.^[^ ^30^, ^36^ ^]^ As a result, achieving controlled 2D Sb_2_S_3_ growth remains an ongoing challenge. An alternative approach to address these challenges is epitaxial growth, which has been widely used for fabricating high‐quality 2D semiconductor thin films, allowing precise thickness control, crystallinity, and lattice registry with the single‐crystalline substrate.^[^ ^37^, ^38^, ^39^ ^]^ Conventionally, epitaxial growth, including molecular beam epitaxy (MBE) and CVD‐based epitaxy, relies on lattice matching between the growing material and the substrate.^[^ ^40^, ^41^ ^]^ However, lattice mismatch and strain accumulation often introduce defects, dislocations, and grain boundaries, which severely limit the quality and scalability of epitaxially grown materials.^[^ ^42^ ^]^ In contrast, template‐assisted growth on vdW surfaces has emerged as a transformative strategy for integrating diverse 2D materials, overcoming the fundamental constraints of lattice mismatch.^[^ ^43^ ^]^ Here, weak vdW interactions rather than strong covalent bonding govern interfacial energetics, enabling strain‐free growth even with lattice‐mismatched systems. When deposited on atomically flat 2D templates such as graphene and TMDs, the reduced diffusion barrier promotes lateral adatom migration and suppresses vertical aggregation, yielding high‐crystallinity 2D films without the constraints of strict lattice matching.^[^ ^44^, ^45^ ^]^ This vdW mediated, templated‐assisted approach has enabled the synthesis of high‐quality 2D heterostructures, quantum dots, and layered chalcogenides, demonstrating its versatility for integrating diverse material systems.^[^ ^44^, ^45^, ^46^ ^]^

In this work, we demonstrate the first synthesis of atomically thin 2D Sb_2_S_3_ nanosheets enabled by vdW template‐assisted growth on monolayer MoS_2_ single crystals at a low growth temperature of 200 °C. Unlike conventional oxide substrates such as SiO_2_, where strong surface interactions impede lateral diffusion and favor the formation of 3D particulates, MoS_2_ template provides an ideal vdW surface that lowers the formation energy barrier and promotes layer‐by‐layer lateral growth. Our theoretical calculations validate this growth mechanism, demonstrating that MoS_2_ surface enhances adatom mobility via reduced diffusion barrier and thus stabilizes the 2D morphology. The resulting Sb_2_S_3_ nanosheets exhibit uniform coverage, atomically sharp interfaces, and sub‐8 nm thickness, with lateral dimensions dictated by the MoS_2_ single‐crystal domain size. Our approach bypasses the thermodynamically favored 1D growth by using low‐temperature, kinetically controlled conditions, where the MoS_2_ vdW template reduces the diffusion barrier and directs lateral 2D growth. Importantly, the planar Sb_2_S_3_/MoS_2_ heterostructures enable enhanced device performance, while achieving an order‐of‐magnitude higher photoresponsivity, broadband detection from ultraviolet to near‐infrared (UV–NIR), and improved field‐effect transistor (FET) characteristics compared to bare MoS_2_. These enhancements are attributed to strong optical absorption and efficient interfacial charge transfer through the high‐quality vdW interface. Beyond Sb_2_S_3,_ this vdW template‐assisted growth strategy holds strong potential for other pnictogen chalcogenides and related 2D material systems, enabling a generalizable pathway to integrate them into atomically thin electronics, optoelectronics, energy storage, and phase‐change memory devices.

## 2 Results and Discussion

**Figure 1:**
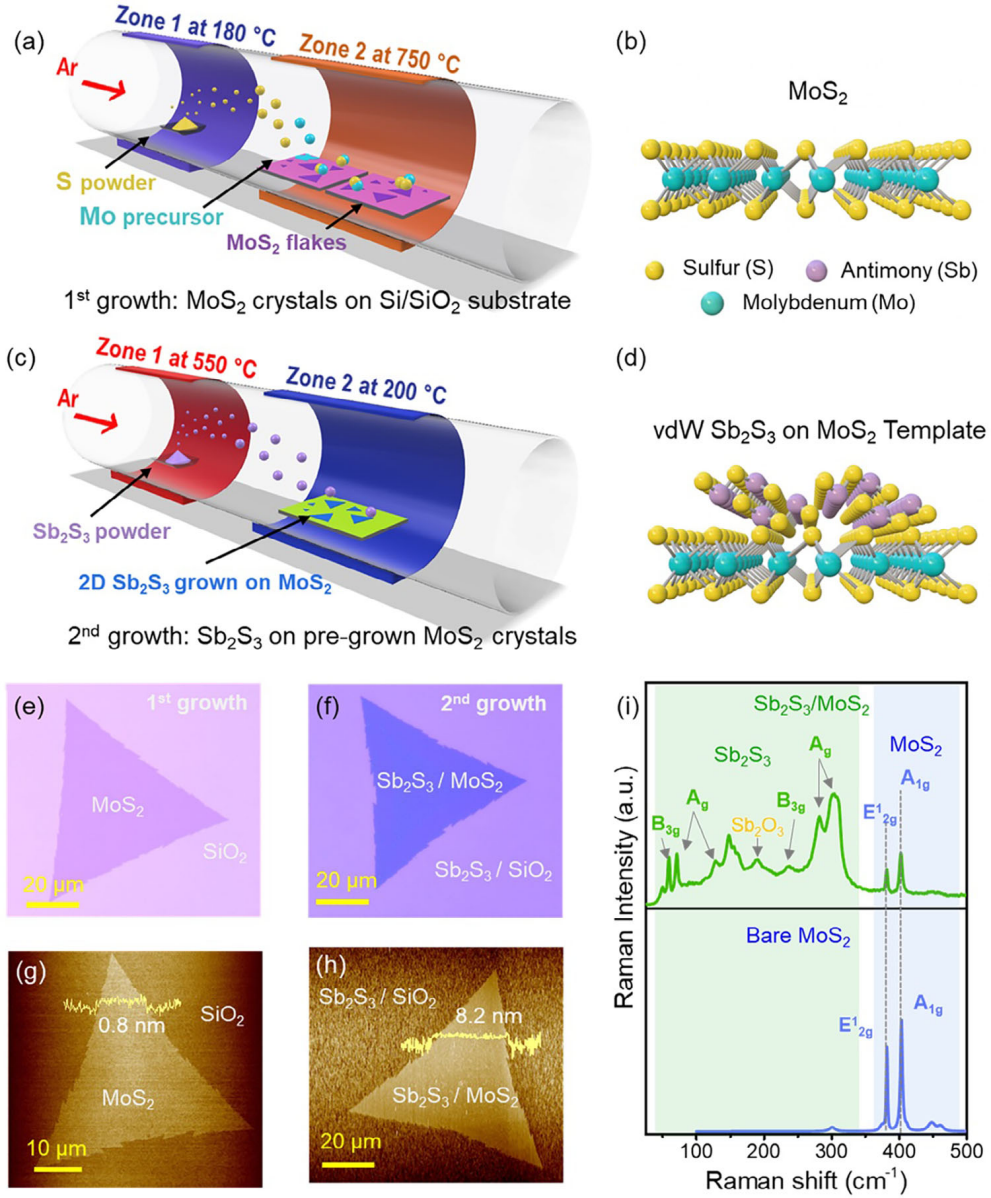
Synthesis and characterization of MoS_2_ and 2D Sb_2_S_3_ on MoS_2_ template. a) Schematic of CVD growth setup of monolayer MoS_2_. b) Atomic model of monolayer MoS_2_ atomic structure c) A schematic of CVD growth setup to grow Sb_2_S_3_ on a pre‐grown monolayer MoS_2_ template. d) Atomic model of Sb_2_S_3_/MoS_2_ stack. e,f) Optical images of a typical as‐grown single crystal MoS_2_ template and the resulting Sb_2_S_3_/MoS_2_ heterostructure samples, respectively. g,h) AFM images of MoS_2_ and Sb_2_S_3_/MoS_2_ heterostructure flakes, respectively. Inset height profiles revealing change in thickness of the monolayer MoS_2_ flake upon 2nd growth of Sb_2_S_3_ i) Raman spectra of bare monolayer MoS_2_ and Sb_2_S_3_/MoS_2_ heterostructure. The A_1g_ and E_2g_ modes correspond to typical monolayer MoS_2_ (labelled in blue). The additional peaks belong to B_3g_ and A_g_ groups corresponding to Sb_2_S_3_(labelled in green).

To achieve the template‐assisted growth of ultrathin 2D Sb_2_S_3_ nanosheets on a monolayer MoS_2_ template, we employed a two‐step CVD process. In the first step, monolayer MoS_2_ single crystals were synthesized using a two‐zone CVD system, as reported in our previous study.^[^ ^47^ ^]^ Briefly, sulfur (S) powder was heated to 180 °C in zone‐1, generating vapors that was carried downstream by argon (Ar) as carrier gas to zone‐2, where 300 nm SiO_2_/Si substrates pre‐coated with Mo‐precursor were placed at 750 °C under low‐pressure conditions, as illustrated in **Figure** **1**a. In Figure 1b, the cross‐sectional atomic model of a typical as‐grown MoS_2_ single‐crystal is depicted. Subsequently, using this single crystal MoS_2_ as vdW seed layer, Sb_2_S_3_ deposition was carried out in the 2nd step of CVD growth, as schematically presented in Figure 1c. In a typical CVD process, Sb_2_S_3_ precursor powder was sublimated at 550 °C in zone‐1, and the resulting vapors were transported by Ar as a carrier gas into zone‐2, where 2D Sb_2_S_3_ nanosheets were deposited on a single‐crystal MoS_2_ template at a much lower temperature of 200 °C. Figure 1d shows the atomic model of the resulting 2D Sb_2_S_3_ on MoS_2_ vdW template, representing the intended vdW‐stacked configuration formed through this sequential growth process. To evaluate the suitability of monolayer MoS_2_ as a vdW template for the lateral growth of 2D Sb_2_S_3_, we conducted preliminary growth experiments by depositing Sb_2_S_3_ on both MoS_2_ single‐crystals and bare SiO_2_/Si substrates. However, the optical images (Figure S1, Supporting Information) revealed that Sb_2_S_3_ adhered preferentially and more uniformly to the MoS_2_ surface compared to bare SiO_2_/Si. This observation suggests that MoS_2_ offers a more favorable surface for Sb_2_S_3_ nucleation and growth, attributed to the vdW interaction between the two materials. Notably, in control experiments at a higher growth temperature of 350 °C, the Sb_2_S_3_ morphology transitioned from lateral 2D sheets to 1D nanorod‐like domains (Figure S2, Supporting Information). This transition reflects the intrinsic thermodynamic preference for anisotropic growth along the [010] direction at higher temperatures, consistent with the quasi‐1D crystal structure of Sb_2_S_3_ and prior observations of 1D Sb_2_S_3_ growth on tungsten disulfide (WS_2_) at 650 °C.^[^ ^48^ ^]^ In contrast, the lower growth temperature (≈200 °C) used in this work establishes non‐thermodynamically conditions that facilitate lateral adatom diffusion on MoS_2_ vdW surfaces, stabilizing the 2D morphology. While higher temperatures can improve crystallinity, they hinder lateral growth, which is essential for forming layered vdW heterostructures with MoS_2_. Based on these insights, we optimized the CVD parameters to achieve the controlled growth of 2D Sb_2_S_3_ on MoS_2_ single‐crystal template. Figure 1e shows the optical image of a representative as‐grown single‐crystal MoS_2_ obtained from the 1st CVD growth, which served as the vdW template for subsequent Sb_2_S_3_ deposition. Following the 2nd CVD step, the optical image of the resulting Sb_2_S_3_ grown on MoS_2_ template is shown in Figure 1f. The clear optical contrast between the monolayer MoS_2_ and the Sb_2_S_3_/MoS_2_ stack on 300 nm SiO_2_/Si substrate highlights the difference in flake thickness, confirming the successful synthesis of Sb_2_S_3_ on MoS_2_ single‐crystal template.

To gain quantitative insight into the thickness variation across the heterostructure, we conducted atomic force microscopy (AFM) analysis following the second CVD step. The AFM image shown in Figure 1g reveals a uniform thickness of ≈0.8 nm of as‐grown MoS_2_ single crystal domain, consistent with a CVD grown monolayer MoS_2_ films.^[^ ^49^ ^]^However, following the deposition of Sb_2_S_3_, the AFM image shown in Figure 1h reveals an increased thickness of ≈8.2 nm across the Sb_2_S_3_/MoS_2_ stack, confirming the formation of a Sb_2_S_3_ film of ≈7.4 nm thickness. Notably, the AFM line‐scan reflects that the Sb_2_S_3_ grows uniformly on the MoS_2_ surface, whereas on adjacent SiO_2_ regions, the deposition is rough and non‐uniform, with randomly distributed 3D particulates indicating uncontrolled nucleation. This preferential growth behavior is further corroborated by scanning electron microscopy (SEM) imaging (Figure S3, Supporting Information), which reveals quasi‐continuous Sb_2_S_3_ film, formed by laterally coalesced domains on the MoS_2_ template and random 3D particulate deposition on bare SiO_2_. These observations highlight the role of MoS_2_ as a vdW template, where its atomically flat surface facilitates lateral adatom diffusion and ordered nucleation of Sb_2_S_3_ via interfacial vdW interactions (discussed later). Furthermore, we found that the thickness of 2D Sb_2_S_3_ can be systematically tuned by adjusting the duration of the second CVD growth, achieving isolated flakes as thin as ≈5.8 nm (Figure S4, Supporting Information). While prolonged growth for 30 min yielded quasi‐continuous Sb_2_S_3_ films with a thickness of ≈28.6 nm (Figure S5, Supporting Information). For all subsequent characterization and device fabrication in this study, we focused on ultrathin films (≈8 nm) to ensure consistency and maximize performance in atomically thin heterostructure devices.

To assess the structural evolution and interfacial coupling in the vdW heterostructure, we carried out Raman spectroscopy on both monolayer MoS_2_ and the resulting Sb_2_S_3_ films grown on MoS_2_. As shown in Figure 1i, the Raman spectrum of monolayer MoS_2_ displays two prominent peaks: the E^1^_2g_ mode (≈382 cm^−1^) corresponding to in‐plane vibrations, and the A_1g_ mode (≈404 cm^−1^) associated with out‐of‐plane vibrations of Mo–S atoms within the lattice structure.^[^ ^49^ ^]^ These peaks exhibit a typical frequency separation (Δ) of ≈21 cm^−1^, consistent with our previous LPCVD grown monolayer MoS_2_ with S vacancy defects.^[^ ^49^ ^]^ Following Sb_2_S_3_ deposition, the Raman spectrum of MoS_2_ reveals a slight red shift in the A_1g_ mode from 404 to 403 cm^−1^, while the E_2g_ mode remains unchanged at ≈382 cm^−1^. This red shift in A_1g_ is attributed to n‐type doping in MoS_2_ induced by the Sb_3_S_3_ overlayer, consistent with previous studies and corroborated by our FET measurements (discussed later), which show increased electron concentration. In addition, to further probe the spectral features, peak deconvolution was performed(Figure S6, Supporting Information), which revealed broadening of the peaks, indicating a modification in the phonon environment and bonding dynamics at the interface.^[^ ^50^ ^]^ The shift and broadening are likely arising from interlayer interactions, strain, or charge transfer effects induced during the Sb_2_S_3_/MoS_2_ heterostructure formation.^[^ ^51^ ^]^ The broadening of the E_2g_ mode, which is attributed to inhomogeneous tensile strain resulting from the lattice mismatch between Sb_2_S_3_ and MoS_2,_ which is consistent with strain modulation commonly observed in vdW heterostructures. Typically, the high‐temperature CVD growth promotes strong interfacial contact that helps retain this strain, thereby contributing to the observed phonon broadening.^[^ ^52^ ^]^ Nevertheless, the more pronounced shift in the A_1g_ mode suggests that out‐of‐plane vibrations are more strongly affected by the overlayer, consistent with vdW coupling at the heterointerface.^[^ ^53^ ^]^ In addition, weak shoulder features near 375 and 408 cm^−1^, corresponding to LO(M) and ZO(M) modes, respectively, are evident. These features suggest the presence of S vacancy‐induced or interfacial defects, in line with our previous work on defect‐engineered CVD‐grown MoS_2_.^[^ ^47^ ^,^ ^54^Importantly, our high‐resolution transmission electron microscopy (HRTEM) and selected area electron diffraction (SAED) analyses (discussed later) confirm that the underlying MoS_2_ retains its single‐crystalline nature after Sb_2_S_3_ growth, demonstrating that the template‐assisted growth process preserves the structural integrity of the MoS_2_ template. Beyond the MoS_2_ modes, the Raman spectrum of the Sb_2_S_3_/MoS_2_ heterostructure exhibits multiple peaks(Figure 1i) at ≈50, ≈60, ≈71, ≈129, ≈148, ≈238, ≈282, and ≈302 cm^−1^, which can be assigned to A_g_ and B_3g_ vibrational modes of Sb–S bonds, consistent with the orthorhombic *Pnma* phase of Sb_2_S_3_.^[^ ^54^ ^]^ Furthermore, our preliminary polarized Raman of the ≈282 cm^−1^ mode shows angular variation (Figure S7, Supporting Information), suggesting anisotropic vibrational behavior consistent with orthorhombic symmetry. In the as‐grown samples, Raman peaks appear broadened (Figure S8, Supporting Information), which we attribute to lattice disorder and interfacial strain introduced during the initial CVD growth. However, following mild annealing at 300 °C in an N_2_ environment, these peaks become sharper and more clearly resolved. A weaker peak at ≈188 cm^−1^ is assigned to α‐Sb_2_O_3_, likely originating from laser‐induced photo‐oxidation of Sb_2_S_3_ in ambient conditions, which is an effect commonly observed due to the chemical instability of the stibnite phase.^[^ ^50^, ^55^ ^]^

**Figure 2:**
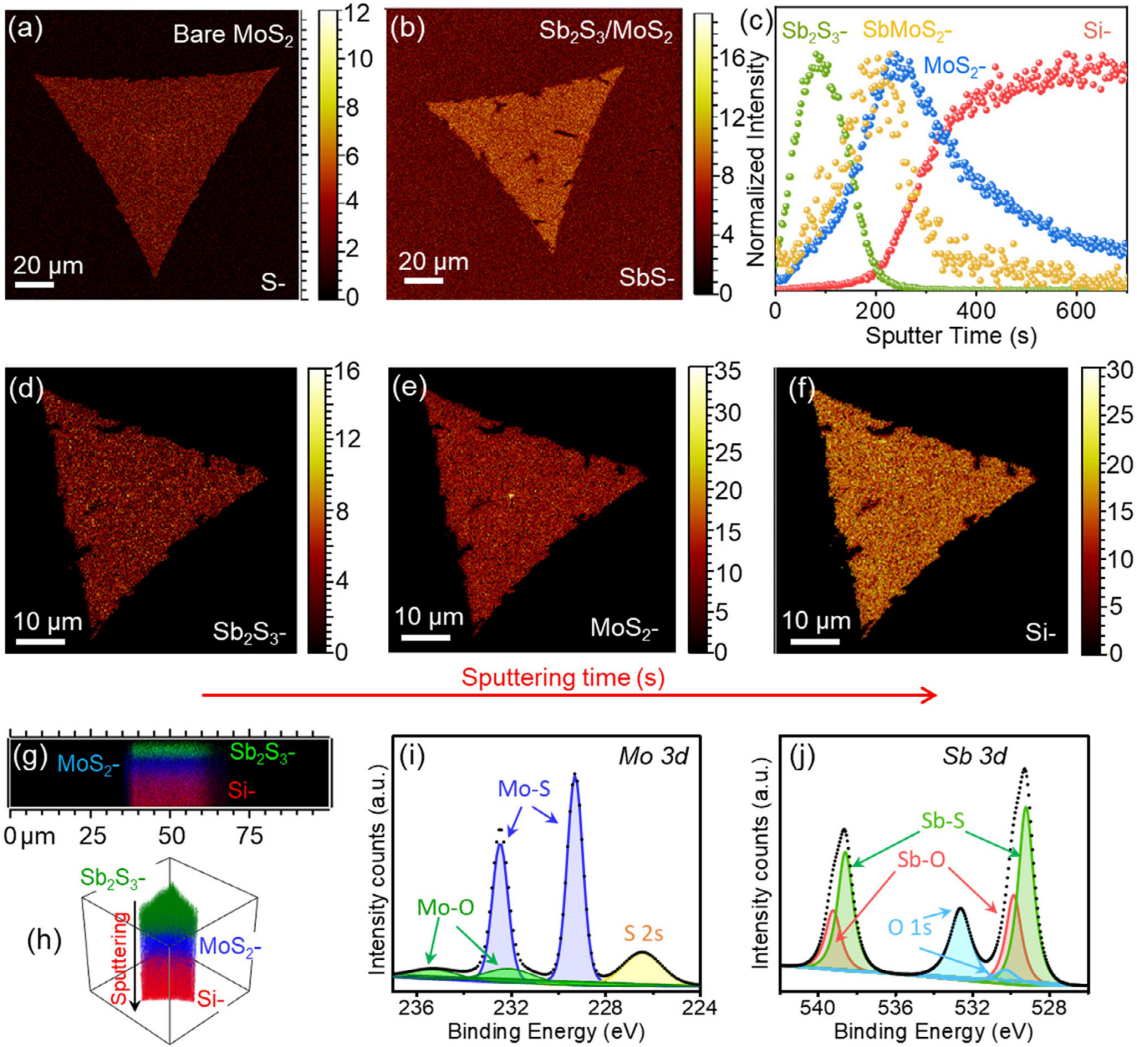
Chemical compositional analysis of 2D Sb_2_S_3_ grown on MoS_2_ vdW template a,b) ToF‐SIMS high lateral resolution surface map of S‐ and SbS‐ secondary ions representing as‐grown MoS_2_ crystal and Sb_2_S_3_/MoS_2_ stacked sample. The distribution of chemical maps is consistent with optical images of triangular single crystals. c) Depth profile of Sb_2_S_3_‐, SbMoS_2_‐, MoS_2_‐ and Si‐ ions against sputtering time of Sb_2_S_3_/MoS_2_ stack grown on SiO_2_/Si substrate.d–f) Sb_2_S_3_^–^, MoS_2_‐ and Si‐ ion map accumulated from depth profiling after a certain time of Cs^+^ sputtering, unveiling the Sb_2_S_3_, MoS_2,_ and SiO_2_ surfaces underneath, respectively. g) 2D and h) 3D cross‐section view of Sb_2_S_3_^–^, MoS_2_‐ and Si‐ ion distribution in the z‐axis obtained from mapping xz and yz slices. The yz cross‐section reveals the stacking of the Sb_2_S_3_/MoS_2_ heterostructure on SiO_2_/Si substrate. i,j) XPS spectra of the Mo 3d and Sb 3d obtained for as‐grown MoS_2_ and Sb_2_S_3_ grown on MoS_2_ template, showing characteristic peaks of Mo^4+^ and Sb^3+^ 3d doublets, respectively. Also shows S 2s associated with MoS_2_ and Sb_2_S_3_.

To further evaluate the chemical composition and vertical stacking of the as‐grown MoS_2_ and Sb_2_S_3_/MoS_2_ heterostructure samples, we employed a combination of Time‐of‐Flight Secondary Ion Mass Spectrometry (ToF‐SIMS) and X‐ray Photoelectron Spectroscopy (XPS). ToF‐SIMS 3D analysis (depth profiling and imaging) is particularly well‐suited for analyzing 2D heterostructures, as it provides spatially resolved chemical information both at the surface and along the depth of the material.^[^ ^56^ ^]^ **Figure** **2**a,b displays the ToF‐SIMS surface maps of S^−^ and SbS^−^ ions, corresponding to monolayer MoS_2_ and Sb_2_S_3_ grown on MoS_2_ template, respectively. The observed triangular features in both maps closely match the single‐crystal shapes seen in the optical images (Figure 1e,f), confirming the spatial correlation between the optical and chemical signatures. To further resolve the vertical composition, we performed ToF‐SIMS depth profiling using Cs^+^ sputtering on the Sb_2_S_3_/MoS_2_ stacked sample. As shown in Figure 2c, the initial increase in Sb_2_S_3_^−^ signal indicates the presence of the Sb_2_S_3_ top layer. After ≈80 s of sputtering, the Sb_2_S_3_‐ intensity begins to decrease, coinciding with a sharp rise in MoS_2_^−^ signal, confirming the underlying MoS_2_ layer. Continued sputtering results in a decline of MoS_2_^−^ signal and a subsequent rise in Si^−^ ion intensity, corresponding to the Si/SiO_2_ substrate beneath the heterostructure. These depth‐resolved chemical profiles unambiguously validate the vertical stacking sequence of the heterostructure, with Sb_2_S_3_ on top of MoS_2,_ and demonstrate the layer‐specific distribution of chemical species across the interface. Moreover, a mixed ion MoSbS_2_^−^ associating with Sb_2_S_3_ and MoS_2_ was found at the interface of the heterostructure, which evidences the interaction between Sb_2_S_3_ and MoS_2_. Figure 2d–f present the cumulative ion maps of Sb_2_S_3_^−^, MoS_2_^−^, and Si^−^, integrated over the full sputtering duration (0–700 s), respectively, depicting the elemental distribution across the vertical depth of the Sb_2_S_3_/MoS_2_ stack. To visualize the spatial arrangement of these species along the z‐axis (depth direction), we extracted cross‐sectional slices from both the horizontal (xz) and vertical (yz) planes across a representative single‐crystal domain. As shown in Figure 2g, the yz cross‐sectional map reveals a well‐defined vertical sequence: Sb_2_S_3_‐ ions are concentrated near the surface, followed by a distinct layer of MoS_2_‐ ions, and finally, Si‐ signals corresponding to the SiO_2_/Si substrate. This sequence confirms the vertical stacking order of the structure, with Sb_2_S_3_ grown atop MoS_2_, as synthesized by vdW template‐assisted growth. Furthermore, the 3D ions distribution rendering (Figure 2h), provides a comprehensive visualization of the chemical architecture in vertical direction, clearly outlining the layered arrangement of Sb_2_S_3_, MoS_2_, and the substrate, and consistent with the ToF‐SIMS depth profile (Figure 2c), reinforcing the chemical and spatial fidelity of the obtained heterostructure.

To corroborate these insights from ToF‐SIMS analysis, we further investigated the chemical states and bonding configurations in the MoS_2_ and Sb_2_S_3_ nanosheets on MoS_2_ template using XPS measurements. The high‐resolution XPS spectrum of the bare MoS_2_ sample is shown in Figure 2i, where clear peaks from the Mo 3d and S 2s orbitals are observed. The dominant peaks at 232.51 and 229.31 eV are assigned to Mo 3d_3/2_ and Mo 3d_5/2_ orbitals of Mo^4+^, respectively, while the peak at 226.41 eV corresponds to S 2s core level, confirming the presence of chemically bonded Mo and S species consistent with MoS_2_.^[^ ^49^ ^]^ In addition to these main peaks, weaker doublets centered at approximately 232 and 235 eV suggest the presence of oxidized Mo species (MoO_x_), which likely arise from minor Mo–O bonding due to ambient air exposure.^[^ ^47^ ^]^ In contrast, the Mo 3d signal is largely suppressed in the XPS spectrum of the Sb_2_S_3_/MoS_2_ stacked sample (Figure S9, Supporting Information), which we attribute to photoelectron attenuation by the overlying Sb_2_S_3_ layer. Nevertheless, the presence and stacking order of MoS_2_ beneath the Sb_2_S_3_ layer are independently validated by Raman spectroscopy and ToF‐SIMS depth profiling (Figure 2c,g,h), which confirm the layered stacking order and spatial continuity of the heterostructure. The corresponding Sb 3d spectrum for the Sb_2_S_3_/MoS_2_ heterostructure (Figure 2j) shows distinct peaks at 529.3 eV (Sb 3d_5/2_) and 538.6 eV (Sb 3d_3/2_), attributed to Sb^3+^ in Sb_2_S_3_, confirming the formation of the intended orthorhombic phase. Quantitative analysis yields an Sb 3d (Sb_2_S_3_) to S 2s signal ratio of ≈0.4, in excellent agreement with the ideal 2:3 stoichiometry.^[^ ^57^ ^]^ A weaker secondary Sb 3d doublet at 529.9 eV (Sb 3d_5/2_) and 539.3 eV (Sb 3d_3/2_) corresponds to Sb_2_O_3._ Based on the inelastic mean free path of Sb 3d electrons (1.9 nm for Sb_2_S_3_ and 1.8 nm for Sb_2_O_3_), the surface oxide overlayer thickness is estimated to be ≈0.7 nm (accounting for ≈33% of the total Sb signal).^[^ ^58^ ^]^ This surface oxide layer is consistent with the minor oxide peak in our Raman spectra and is attributed to the known chemical instability of Sb_2_S_3_ in ambient oxygen, reported previously.^[^ ^55^ ^]^ Collectively, the complementary results from ToF‐SIMS, and quantitative XPS confirm that the overlayer is predominantly stoichiometric Sb_2_S_3_ with slight surface oxidation arising from ambient exposure.

**Figure 3:**
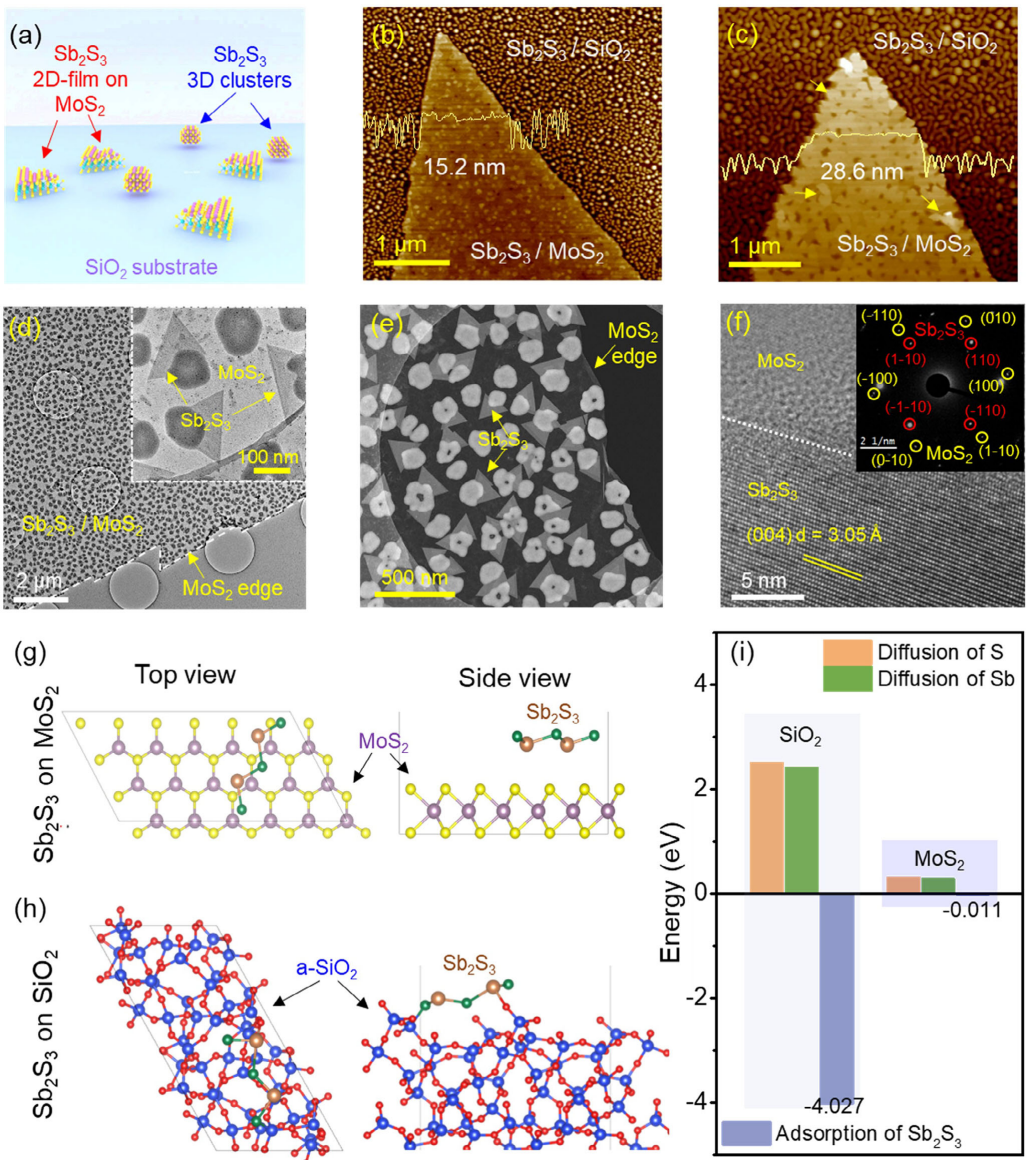
Mechanism of vdW template‐assisted growth of Sb_2_S_3_ on MoS_2_. a) Schematic illustration of lateral 2D Sb_2_S_3_ growth on MoS_2_ surfaces contrasted with uncontrolled Sb_2_S_3_ cluster nucleation on SiO_2_ surface. b,c) High‐resolution AFM scan of Sb_2_S_3_ film grown on MoS_2_ template with in‐set height profiles unveiling the quasi‐continuous 2D Sb_2_S_3_ film formation on MoS_2_ and Sb_2_S_3_ particulates on SiO_2_ surface. d) Low‐magnification TEM image revealing triangular Sb_2_S_3_ flakes and amorphous seed‐like features on MoS_2_ (inset: high magnification view). e) Annular dark‐field STEM image showing randomly oriented triangular Sb_2_S_3_ flakes on MoS_2_. f) Atomic resolution TEM image of the Sb_2_S_3_/MoS_2_ showing (004) planes of Sb_2_S_3_ (d = 3.05 Å). SAED pattern (inset) displays diffraction spots from orthorhombic Sb_2_S_3_ and hexagonal MoS_2_. g,h) Atomic structural model obtained from DFT showing adsorption of Sb_2_S_3_ on MoS_2_ and SiO_2,_ respectively. i) DFT energy profiles for diffusion of S and Sb atoms and adsorption of Sb_2_S_3_ molecules on amorphous SiO_2_ and MoS_2_ vdW surface, respectively.

To gain deeper insight into the mechanism driving the preferential lateral growth of 2D Sb_2_S_3_ on MoS_2_ template, we examined the morphological evolution at the growth interface using high‐resolution AFM and TEM analyses. As illustrated in **Figure** **3**a, the vdW surface of monolayer MoS_2_ plays a decisive role in directing the lateral 2D growth of 2D Sb_2_S_3_, in contrast to the randomly oriented 3D particulates observed on bare SiO_2_. High‐resolution AFM images (**Figure** **3**b,c) of Sb_2_S_3_ grown on MoS_2_ single‐crystal template supported on SiO_2_/Si substrates enable a direct comparison of growth behavior across the two surfaces. The AFM scans, along with the corresponding line profiles (insets), clearly show that Sb_2_S_3_ grown on MoS_2_ is atomically smooth and has uniform coverage, indicative of layered growth. In contrast, Sb_2_S_3_ nucleated on bare SiO_2_ exhibits discrete, randomly distributed 3D clusters, as evidenced by the rough and irregular height profile, and Raman spectra acquired in this region shows no peaks corresponding to Sb_2_S_3_ (Figure S10, Supporting Information). Interestingly, at longer growth durations, the Sb_2_S_3_ films formed on MoS_2_ become thicker yet remain flat, forming well‐defined 2D nanosheets with lateral dimensions of several tens of nanometers, as shown in Figure 3c. This evolution underscores the stability of the Sb_2_S_3_ layered morphology and further validates a template‐driven 2D growth mechanism, enabled by the vdW surface of MoS_2_. To directly probe the atomic structure and crystallinity of 2D Sb_2_S_3_ grown on MoS_2_, we performed HRTEM analysis on Sb_2_S_3_/MoS_2_ stacks transferred directly from the growth substrate to the holey carbon‐coated Cu TEM grids.^[^ ^59^ ^]^ The low‐magnification TEM image (Figure 3d) reveals a uniform distribution of well‐faceted triangular Sb_2_S_3_ flakes across the underlying MoS_2_ domains. Alongside these flakes, darker diffuse regions are visible, which we attribute to the initial nucleation of amorphous Sb_2_S_3_ seeds formed during the early growth stage, indicative of a nucleation–growth transition from amorphous clusters to well‐defined crystalline domains. The corresponding annular dark‐field STEM image (Figure 3e), together with energy‐dispersive X‐ray spectroscopy (EDS) mapping (Figure S11, Supporting Information), further confirms the presence of discrete triangular Sb_2_S_3_ flakes, randomly oriented on the MoS_2_ surface. High‐resolution TEM imaging (Figure 3f) shows clear lattice fringes with a measured interplanar spacing of 3.05 Å, corresponding to the (004) planes of orthorhombic Sb_2_S_3_. The selected‐area electron diffraction (SAED) pattern from the same region exhibits diffraction spots indexed to orthorhombic Sb_2_S_3_ ((110), (1–10), (–110), (–1–10)) alongside the six‐fold symmetric spots of hexagonal MoS_2_, confirming the stacking of Sb_2_S_3_ crystals on MoS_2_ template. Importantly, the sharp diffraction spots inherent to single‐crystal MoS_2_ (Figure S12, Supporting Information) indicate that the underlying single‐crystal remains structurally intact after Sb_2_S_3_ growth, demonstrating preservation of the MoS_2_ lattice during the second growth step. Notably, SAED analysis of multiple flakes indicates that Sb_2_S_3_ adopts arbitrary in‐plane orientations relative to MoS_2_. The absence of any preferred alignment confirms the lack of a strictly epitaxial relationship, given the crystallographic incompatibility between hexagonal MoS_2_ and orthorhombic Sb_2_S_3_ lattices. Instead, the appearance of laterally oriented triangular Sb_2_S_3_ flakes points to a, template‐assisted growth process, in which vdW interactions on the atomically flat MoS_2_ surface suppress the intrinsic chain‐growth tendency and promote lateral growth governed by surface energy minimization rather than lattice matching.

To validate our experimental observations and gain deeper insight into the growth kinetics and interfacial energetics governing the selective lateral growth of 2D Sb_2_S_3_, we performed a theoretical investigation including DFT, comparing its behavior on MoS_2_ and amorphous SiO_2_ surfaces. As shown in Figure 3g–i, we calculated the diffusion energy barriers for S atoms and Sb atoms, which are critical constituents in Sb_2_S_3_ formation on both surfaces. Our DFT calculations reveal a stark contrast in adatom mobility on MoS_2_ versus the bare SiO_2_ surface, such as S and Sb atoms exhibit low diffusion energy barriers of 0.3 and 0.275 eV, respectively, whereas on SiO_2_, the corresponding values rise sharply to 2.5 and 2.41 eV (Figure 3i). These results confirm that the MoS_2_ surface provides a favorable pathway for surface diffusion, promoting efficient lateral adatom migration, promoting layered growth of Sb_2_S_3_, which is consistent with the smooth, uniform 2D films observed in AFM and TEM analysis for 2D Sb_2_S_3_/MoS_2_ heterostructure. In addition to diffusion kinetics, adsorption energy (E_ads_) calculations further elucidate the difference in interfacial interaction. The E_ads_ of an Sb_2_S_3_ molecule on MoS_2_ is –0.011 eV, indicating weak physisorption governed by vdW interactions, allowing the precursors to diffuse freely. In contrast, the adsorption of Sb_2_S_3_ on amorphous SiO_2_ is much stronger (E_ads_ = –4.027 eV), indicating strong chemisorption, which traps adatoms and impedes their mobility. This explains the formation of 3D clusters and amorphous domains observed on SiO_2_ in the absence of a vdW template. Collectively, these DFT results provide a thermodynamic and kinetic rationale for the experimentally observed selective lateral growth of 2D Sb_2_S_3_ on MoS_2_. The MoS_2_ vdW surfaces lowers the energy barriers, enhances adatom mobility, and promotes ordered 2D growth, in contrast to oxide substrate (non‐vdW surfaces) that that trap adatoms due to stronger, immobilizing interactions leading to random 3D nucleation. Furthermore, the versatility of vdW template‐assisted approach is demonstrated by the successful growth of 2D Sb_2_S_3_ on monolayer WS_2_ single crystals, yielding uniform coverage and distinct Raman modes from both constituents (Figure S13, Supporting Information), confirming its applicability across a broader range of TMD templates beyond MoS_2_.

**Figure 4:**
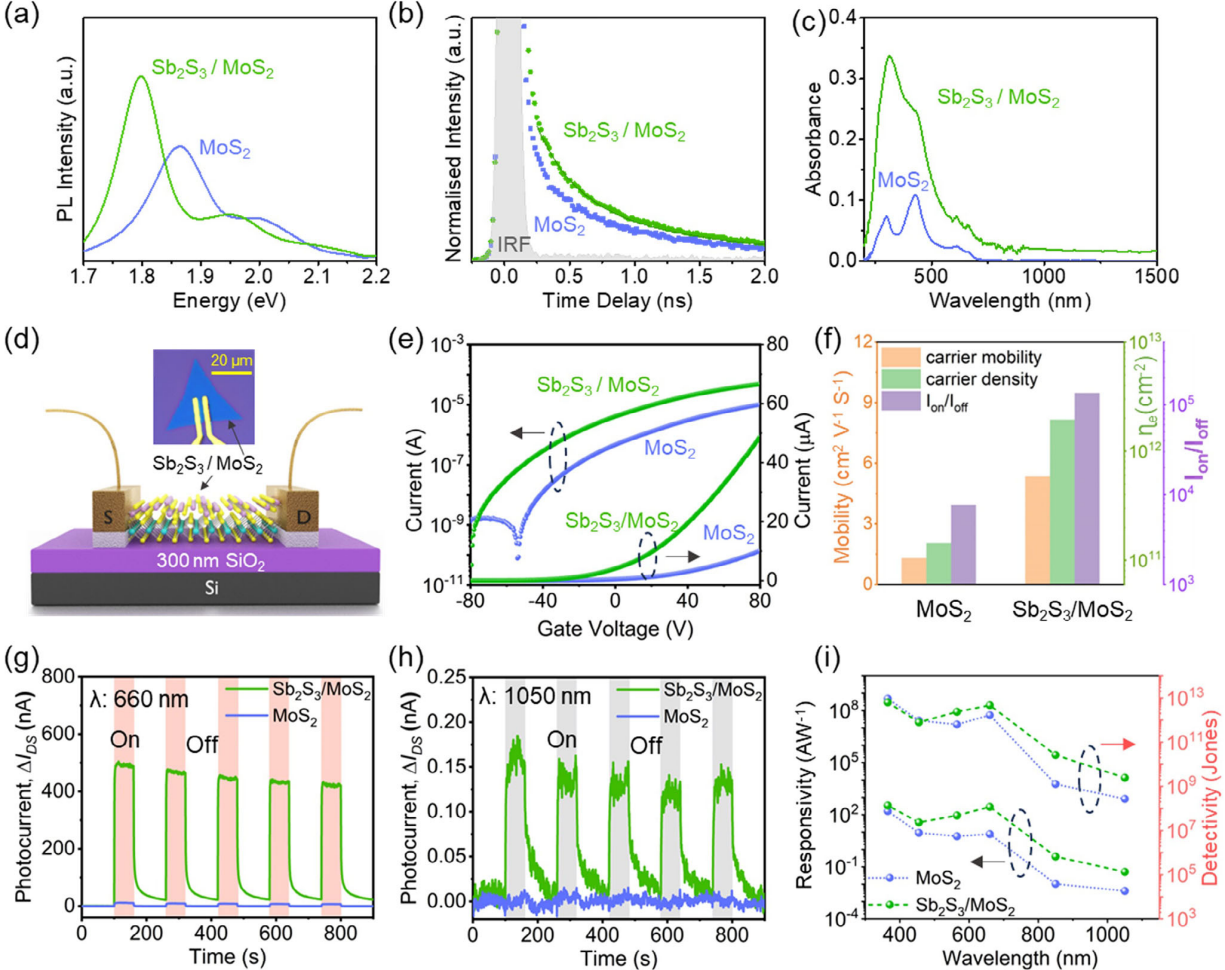
Optical, electronic, and optoelectronic characterization a) PL spectra reveal a decrease in bandgap in Sb_2_S_3_/MoS_2_ heterostructure compared to only monolayer MoS_2_ b) Time‐resolved PL measurements showing longer decay lifetimes for Sb_2_S_3_/MoS_2_ sample than MoS_2_ only sample. c) UV–visible‐IR light absorbance spectrum showing higher and broader range absorption in the heterostructure than bare MoS_2_. d) 3D schematic of Sb_2_S_3_/MoS_2_ heterostructure on 300 nm SiO_2_/Si substrate. The in‐set shows the optical image of a representative fabricated device. The channel length is ≈ 3 µm. e) Transfer characteristics of MoS_2_ and Sb_2_S_3_/MoS_2_ based FET, with current on a log and linear scale f) comparison of field‐effect electron mobility, carrier density, and I_on_/I_off_ for MoS_2_ and Sb_2_S_3_/MoS_2_ FETs g,h) Time‐based photo‐response measurements of MoS_2_ and Sb_2_S_3_/MoS_2_ photodetectors under 660 and 1050 nm illumination, 4 mW cm^−2^ power density and at constant V_ds_ of 2 V i) Photoresponsivity and detectivity of MoS_2_ and vdW surface enabled grown Sb_2_S_3_/MoS_2_ stack, respectively.

With the successful synthesis and structural verification of template‐assisted 2D Sb_2_S_3_ growth on MoS_2_, we next evaluated their intrinsic optical and electronic properties and explored functional potential for optoelectronic and electronic device applications. We acquired room temperature photoluminescence (PL) spectra from monolayer MoS_2_ and Sb_2_S_3_/MoS_2_ heterostructure samples, shown in **Figure** **4**a. The PL spectrum of monolayer MoS_2_, exhibits a strong emission peak at ≈1.86 eV, which corresponds to A exciton, and a weaker excitonic peak at 2.01 eV associated with B exciton, arising from direct bandgap transition and spin–orbit‐induced valence band splitting, respectively.^[^ ^49^ ^]^ Interestingly, following Sb_2_S_3_ deposition, the PL spectrum of the Sb_2_S_3_/MoS_2_ flakes exhibits a red shift in both peaks to ≈1.79 eV (exciton‐A) and ≈1.96 eV (exciton‐B), accompanied by an enhancement in the PL intensity, suggesting an interlayer electronic coupling.^[^ ^60^ ^]^ This behavior suggests interfacial charge transfer between Sb_2_S_3_ and MoS_2_ layers. To investigate this further, time‐resolved PL (TRPL) measurements were performed to observe the decay lifetime of excitons, as presented in Figure 4b. A bi‐exponential decay model was used to extract the fast (*τ*_1_) and slow (*τ*_2_) components of exciton recombination in both monolayer MoS_2_ and Sb_2_S_3_/MoS_2_ heterostructure samples. The fast decay (*τ*_1_ ≈0.3 ns) remained unchanged across both samples, while the slow component (*τ*_2_) increased slightly from 1.31 ns in MoS_2_ to 1.40 ns in the heterostructure, suggesting modest suppression of non‐radiative recombination and evidence of interfacial charge transfer dynamics. Figure 4c presents in the absorbance spectra acquired from monolayer MoS_2_ and Sb_2_S_3_/MoS_2_ stack samples, further supporting these findings of modifications in optical behavior following Sb_2_S_3_ deposition. Monolayer MoS_2_ exhibits typical excitonic absorption peaks at ≈610 nm (≈2.02 eV) and ≈658 nm (≈1.88 eV), consistent with the A and B excitons observed in the respective PL spectrum. In the Sb_2_S_3_/ MoS_2_ stack samples, these features are not only retained but also significantly enhanced, confirming improved visible light absorption. Importantly, while the absorbance of bare MoS_2_ declines sharply beyond 750 nm, the heterostructure retains substantial absorption well into the near‐infrared (NIR) region, indicative of an extended spectral photoresponse enabled by Sb_2_S_3_, which we observed (as discussed in the next section). Additional absorbance enhancement is also observed in the UV region (<500 nm), where Sb_2_S_3_ dominates due to its high absorption coefficient in the UV range.^[^ ^31^ ^]^ These results demonstrate that the integration of Sb_2_S_3_ onto MoS_2_ not only preserves the inherent excitonic features of MoS_2_ but also broadens and amplifies the optical response, effectively extending the operational window from UV–visible in pristine MoS_2_ to UV–visible–NIR in the heterostructure. Moreover, the Sb_2_S_3_/ MoS_2_ heterostructure is expected to form a type‐II band alignment (Figure S14, Supporting Information),^[^ ^57^ ^]^ which facilitates efficient spatial separation of charge carriers, enabling the extraction of lower‐energy photons and supporting enhanced device performance based on this template‐assisted grown Sb_2_S_3_/ MoS_2_ heterostructure.

To demonstrate the functional potential of the vdW template‐assisted grown 2D Sb_2_S_3_/MoS_2_ heterostructure for electronic and optoelectronic device applications, we fabricated devices directly on the as‐grown samples. Maskless photolithography was employed to define metal contacts and evaluate both the electronic and optoelectronic performance of the heterostructure, with monolayer MoS_2_ devices used as reference. Figure 4d shows the 3D schematic and corresponding optical micrograph (inset) of a representative Sb_2_S_3_/MoS_2_ device fabricated on a 300 nm SiO_2_/Si substrate. The devices utilize lateral Ni/Au top contacts as source and drain, enabling both configurations for operating as two‐terminal photodetectors and three‐terminal FETs, using the highly doped Si (p^++^) as a global back gate. We first evaluated the electrical performance of Sb_2_S_3_/MoS_2_ devices as compared to MoS_2_‐based FETs, as shown in Figure 4e,f. The transfer characteristics (Figure 4e) reveal a marked improvement in the on‐state current (I_o_) for epitaxial grown Sb_2_S_3_/MoS_2_ devices compared to pristine MoS_2_ based FETs, indicating enhanced channel conductivity. Furthermore, both devices exhibit n‐type behavior with negative threshold voltages (V_th_); however, the Sb_2_S_3_/MoS_2_ device shows a larger shift (V_th_ ≈–57 V) compared to the monolayer MoS_2_ device (V ≈–32 V), suggesting a higher degree of n‐doping induced in the heterostructure. This threshold shift is attributed to interfacial charge transfer in the vdW‐stacked 2D Sb_2_S_3_/MoS_2_ heterostructure, which increases the intrinsic carrier concentration. As supported by our calculations (see Supporting Information Section on page 10 for further details), the field‐effect carrier density (*n*_e_) in the Sb_2_S_3_/MoS_2_ device reaches 1.94 × 10^12 ^cm^−2^, which is an order of magnitude higher than that of the MoS_2_ device (*n*_e _ =  1.44 × 10^11 ^cm^−2^), as shown in Figure 4f. Furthermore, the Sb_2_S_3_/MoS_2_ devices exhibit a significantly improved field‐effect mobility of≈ 5 cm^2^ V^−1^ s^−1^, compared to ≈1.2 cm^2^ V^−1^ s^−1^ for pristine monolayer MoS_2_ FETs (Figure 4f). This enhancement arises primarily from efficient interfacial charge transfer. The type‐II band alignment between Sb_2_S_3_ and MoS_2_ facilitates rapid electron–hole separation and generates a built‐in electric field that screens Coulomb scattering from charged impurities and mitigates surface‐related scattering in MoS_2_.^[^ ^48^ ^]^ Such interlayer charge‐transfer‐induced screening, as reported in 2D heterostructures,^[^ ^61^ ^]^ enables higher mobility even in the presence of increased carrier concentration. Furthermore, the output current‐voltage (*I*_DS_‐*V*_DS_) characteristics measured at room temperature (Figure S15, Supporting Information), exhibit linear ohmic behavior for both device types, consistent with our previous studies on monolayer MoS_2_ FETs. This ohmic response indicates low contact resistance and a small Schottky barrier height (SBH) at the Ni/Au–semiconductor interface.^[^ ^62^ ^]^ Therefore, in Sb_2_S_3_/MoS_2_ devices, the combined effects of interlayer charge‐transfer‐induced screening, reduced SBH, and improved interfacial charge injection lead to enhanced on‐state current (I_on_) and field‐effect mobility, confirming that vdW surfaces integration of Sb_2_S_3_ with monolayer MoS_2_ is an effective strategy for modulating and boosting the electronic transport properties of potential atomically thin FET platforms.

The combination of broadband optical absorption and favorable interfacial charge dynamics makes the Sb_2_S_3_/MoS_2_ hybrid structure a highly promising platform for advanced optoelectronic applications, particularly broadband photodetectors. To evaluate its potential as a photodetector, we performed time‐resolved photocurrent measurements under illumination at multiple wavelengths spanning the UV–visible–NIR spectrum (365, 455, 565, 660, 850, and 1050 nm), using a constant LED power density of 4 mW cm^−2^ and a fixed readout voltage of 2 V (See Figure S16, Supporting Information for all wavelength). The Sb_2_S_3_/MoS_2_ devices exhibit a clear and consistent increase in photocurrent (I_DS_) across all tested wavelengths, demonstrating broadband photodetection capability. Notably, under 660 nm illumination, the heterostructure device shows an enhancement of approximately two orders of magnitude in photocurrent compared to the MoS_2_‐only device, as shown in Figure 4g. This substantial increase in photoresponse is characteristic of type‐II band alignment,^[^ ^57^ ^]^ where efficient spatial separation of photogenerated electron–hole pairs enhances carrier extraction. The formation of a type‐II heterojunction between Sb_2_S_3_ and MoS_2_ is further supported by the energy band alignment diagram derived from literature‐reported energy levels (see Figure S14, Supporting Information),^[^ ^57^ ^]^ confirming that the conduction and valence band offsets facilitate interlayer charge separation, also consistent with our optical characterization results discussed above. In addition to the enhanced photocurrent, the dark current (I_dark_) of the Sb_2_S_3_/MoS_2_ device is also approximately an order of magnitude higher than that of bare MoS_2_, consistent with the previously observed n‐type doping effect introduced by Sb_2_S_3_. This increased conductivity is attributed to a higher free carrier concentration, corroborating our FET measurements and thus confirming that the Sb_2_S_3_ overlayer introduces interfacial charge transfer that boosts both dark and photo‐induced currents.

To quantitatively compare the performance of these devices as photodetectors, we calculated key figures of merit, including photoresponsivity (R) and specific detectivity (D^*^), across a broad spectral range from UV to NIR, using the standard expressions (Supporting Information for details). Under a constant drain‐source bias of 2 V, monolayer MoS_2_ photodetectors fail to respond to illumination in the NIR region, as shown in Figure 4h. In contrast, the Sb_2_S_3_/MoS_2_ heterostructure devices exhibit strong and consistent photoresponse across the entire tested range, from 365 nm (UV) to 1050 nm (NIR) (Figure S16, Supporting Information), highlighting their broadband detection capability. As shown in Figure 4i, the heterostructure photodetectors exhibit an order of magnitude higher photoresponsivity and a broadened spectral detectivity range compared to devices based on pristine monolayer MoS_2_. This superior performance is attributed to the efficient interfacial charge separation facilitated by the type‐II band alignment at the Sb_2_S_3_/MoS_2_ interface, coupled with the enhanced broadband absorption of the heterostructure, as previously demonstrated in the absorbance spectra (Figure 4c). The absence of NIR response in monolayer MoS_2_ devices is consistent with its limited absorption beyond the visible range, whereas the Sb_2_S_3_/MoS_2_ heterostructure retains significant absorption well into the NIR, which we attribute to mid‐gap states introduced at the interface. These defect‐mediated states enable sub‐bandgap photon absorption, supported by our TRPL results (Figure 4b) showing an increased slow decay component (τ_2_) from 1.31 ns in MoS_2_ to 1.40 ns in the heterostructure. The type‐II band alignment further promotes carrier extraction from these mid‐gap states via a built‐in electric field,^[^ ^48^ ^]^ sustaining the NIR photoresponse up to ≈1050 nm. Together, these results establish 2D Sb_2_S_3_/MoS_2_ heterostructures grown via vdW template‐assisted growth as a versatile and high‐performance material platform that simultaneously enhancing both electronic transport and broadband photodetection, offering strong prospects for integration into next‐generation 2D nanoelectronics and optoelectronic devices.

## 3 Conclusion

In this work, we have demonstrated a vdW template‐assisted growth approach that realizes controlled synthesis of layered 2D Sb_2_S_3_ nanosheets on monolayer MoS_2_ single‐crystals. Unlike conventional oxide surfaces that promote 3D particulate growth due to strong substrate interactions, the vdW surface of MoS_2_ facilitates low‐energy diffusion and layered lateral growth, as supported by experimental observations and DFT calculations. Furthermore, the resulting Sb_2_S_3_/MoS_2_ heterostructure exhibits well‐defined interfaces, large lateral domain sizes, and sub‐8 nm thickness, forming structurally coherent vertical stacks. Since Sb_2_S_3_ coverage directly follows the lateral dimensions of the underlying MoS_2_ single‐crystal domains, our approach is inherently scalable to wafer scale with continuous large‐area MoS_2_ templates. Comprehensive optical and electrical characterizations reveal that vdW integration of Sb_2_S_3_ significantly modulates the properties of MoS_2_, leading to enhanced light absorption from UV to NIR, interfacial charge transfer, and improved carrier transport. FETs based on the heterostructure show higher on‐state current, increased field‐effect mobility, and greater carrier densities compared to pristine MoS_2_. In addition, broadband photodetectors fabricated from the Sb_2_S_3_/MoS_2_ heterostructure exhibit strong responsivity and detectivity across a wide spectral range, enabled by type‐II band alignment and efficient photocarrier separation. In conclusion, this work overcomes the intrinsic 1D/3D growth tendency of Sb_2_S_3_ and establishes a scalable vdW template‐assisted growth strategy for realizing layered 2D pnictogen chalcogenide heterostructures with TMDs. The demonstrated ultrathin Sb_2_S_3_/MoS_2_ platform, with its well‐defined interfaces and enhanced optoelectronic performance, opens new opportunities for future integration into the next generation of nanoelectronics and optoelectronic devices.

## 4 Experimental Section

### CVD Growth of Monolayer MoS_2_ and Sb_2_S_3_/MoS_2_ Heterostructure

Monolayer MoS_2_ single‐crystals were grown using a two‐temperature zone tube furnace in a low‐pressure (≈1 torr) CVD condition. The growth substrates, 300 nm SiO_2_/Si were ultrasonically cleaned by acetone, IPA, and water sequentially, followed by N_2_ blow drying. An aqueous solution of ammonium molybdate tetrahydrate was used as a Mo precursor, and was drop‐casted (5 µl droplet) onto the cleaned SiO_2_/Si substrates, followed by baking at 110 °C for 5 min. The samples were then loaded into zone‐2 of the tube furnace, under the constant flow of 500 sccm Ar for 10 min. 200 mg of sulfur (S) powder was placed in zone‐1 and heated to 180 °C to create S vapors, which were carried downstream into zone‐2 by 70 sccm flow of carrier gas (Ar). The MoS_2_ single crystals were grown at 750 °C for 20 min. For the 2nd growth of Sb_2_S_3_/MoS_2_ heterostructure, the as‐grown MoS_2_ single‐crystal was loaded into zone‐2. Sb_2_S_3_ precursor powder was in zone‐1 and sublimated at 550 °C, and the resulting vapors were transported by Ar as a carrier gas into zone‐2, where 2D Sb_2_S_3_ nanosheets were deposited on a single‐crystal MoS_2_ template at 200 °C with a growth time of 10 min, while maintaining the growth pressure at 1 torr. After growth, the furnace was cooled to room temperature under the 500 sccm flow of Ar.

### Material Characterization

To obtain optical images of the grown samples, a Leica microscope was utilized. For thickness and morphology analysis, Bruker Dimension Icon AFM with ScanAsyst‐air tip was used for measuring the thickness of the as grown samples. To obtain details about the structural properties of the samples, Raman and PL measurements were undertaken using a HORIBA LabRAM Raman spectrometer with a 532 nm laser source. In addition, the UV–vis‐NIR absorbance spectra were obtained using the CRAIC 20/30 UV−vis microspectrophotometer (CRAIC). JEOL JEM‐F200 CFEG TEM with an acceleration voltage of 200 kV was utilised for physical characterisations, i.e., TEM, HRTEM images, and SAED patterns. XPS data was acquired for the analysis of elemental composition of the defect‐enriched and passivated MoS_2_ using a Kratos AXIS Supra XPS spectrometer with 1486.7 eV (Al Kα) X‐ray source.

### ToF‐SIMS Measurements

ToF‐SIMS measurements were carried out with an IONTOF M6 Hybrid SIMS instrument (ION‐TOF GmbH, Münster, Germany). In this work, two types of ToF‐SIMS analyses were performed: 1) high lateral resolution surface chemical mapping and 2) ToF‐SIMS 3D analysis. For surface chemical mapping, a 30 keV Bi_3_^+^ cluster beam was scanned over a specified raster area of typically 150×150 mm^2^ with a pixel resolution of 256×256. The primary ion beam was optimized for the best spatial resolution (≈ 100 nm). For ToF‐SIMS 3D analysis, a 500 V‐Cs+ beam scanning over typically an area of 400×400 mm^2^ with a current of 7 nA was used to sputter through the 2D materials. High spatial resolution images were taken at the center of Cs sputtering with 256×256 raster pixel sizes. The imaged area was typically 120 × 120 mm^2^.

### Device Fabrication

A standard photolithography process was used to fabricate two‐terminal lateral devices, which include source and drain channels with an active area of ≈25 µm^2^. AZ5214E photoresist was spin‐coated onto the sample, and the source/drain electrodes were patterned with a maskless aligner (MLA150—Heidelberg Instruments). AZ400K and H_2_O mixture (1:3) and AZ726MIF developers were used to develop MoS_2_ and Sb_2_S_3_/MoS_2_‐samples, respectively. After the development of patterned devices, 10 nm Ni followed by 100 nm Au metal contacts were evaporated onto the sample via electron beam deposition (PVD75—Kurt J. Lesker) with a base pressure of <5 × 10^−7^ Torr. The device fabrication process was ended by the lift‐off process in acetone to obtain MoS_2_ and Sb_2_S_3_/MoS_2_ devices.

### Electrical and Optoelectronic Measurements

The electrical measurements were performed using a Keithley 4200SCS semiconductor parameter analyzer and an Agilent 2912A source meter.  Optoelectronic measurements were performed using a Keysight B2912A source measurement unit. All measurements for the devices were taken under dark ambient conditions except during optical excitation with uncollimated monochromatic LEDs (from Thorlabs Inc.) with wavelengths of 285, 365, 455, 565, 660, 850, and 1050 nm. A programmable Arduino‐uno microcontroller was used for pulse width modulations of these excitation sources.

### Computational Methods

The optimized adsorption configuration was obtained via density functional theory using the projector augmented wave as implemented in the Vienna Ab initio Simulation Package.^[^ ^63^, ^64^ ^]^ The Sb_2_S_3_ adsorption energy was calculated based on the equation: E_ads_ = E_tot_ – E_surface_ – E_molecule_, where E_tot_, E_surface_, and E_molecule_ correspond to the total energy of the combined system, the total energy of the clean surface, and the total energy of the Sb_2_S_3_ isolated molecule. The Perdew–Burke–Ernzerhof approximation was used for the exchange‐correlation functional.^[^ ^65^ ^]^ The cut‐off energy of the plane‐wave basis was set to 400 eV, and self‐consistent calculations did not stop until energy changes were <1 × 10^−4^ eV. The initial geometries of amorphous SiO_2_ were obtained by performing molecular dynamics calculations on the SiO_2_(001) surface with O bond termination, as previously reported to be the lowest energy facet on SiO_2_.^[^ ^66^ ^]^ The calculations were carried out using the NVT ensemble with a Nose–Hoover thermostat set to a target temperature of 700 K.^[^ ^67^ ^]^ All NVT‐MD calculations were performed at the Γ‐point. Simulation times of 20 ps with a timestep of 1 fs were found to be sufficient for surface reconstructions to take place. The formed new siloxane bridges under coordinated Si cations were chosen to be the diffusion path of S and Sb atoms. The minimum‐energy pathway and diffusion barriers for S and Sb atoms on MoS_2_ and the SiO_2_ surface were obtained from the climbing‐image nudged elastic band method as implemented within the Atomic Simulation Environment package with the FIRE algorithm, and CHGnet was employed as the calculator.^[^ ^68^ ^]^ All structures were fully relaxed until the residual force per atom reached <0.1 eV Å^−1^.

## Conflict of Interest

The authors declare no conflict of interest.

## Supporting information



Supporting Information

## Data Availability

The data that support the findings of this study are available from the corresponding author upon reasonable request.
