# Supplementary material for: Van der Waals Template‐Assisted Growth of Two‐dimensional Sb2S3
Source: Adv Sci (Weinh). 2025 Sep 29;12(46):e09903. doi: 10.1002/advs.202509903 (PMC12697887; doi:10.1002/advs.202509903)
Supplement: Supplementary file 1 — Supporting Information [file ADVS-12-e09903-s001.docx]

**Supplementary Information**

**Van der Waals Template-Assisted Growth of Two-dimensional Sb_2_S_3_**

Sindhu Priya Giridhar,^1,9^ Irfan H. Abidi,^1,9,*^ Jiawen Qiu,^2^ Ghalib Alfaza,^3^ Jonathan O.  Tollerud,^4^ Pargam Vashishtha,^1,5^ Jianfeng Mao,^1^ Edwin LH Mayes,^6^ Billy J. Murdoch,^6^ Mei Xian Low,^1^ Yuxiao Hou,^2^ Taimur Ahmed,^1^ Jeffrey A. Davis,^4^ Enrico Della Gaspera,^7^ Priyank Kumar,^3^ Lu- Tao Weng,^2,8^ Sumeet Walia ^1, *^

*^1^Centre for Opto-electronic Materials and Sensors (COMAS), School of Engineering, RMIT University, 124 La Trobe Street, Melbourne, Victoria 3001, Australia.*

*^2^Materials Characterization and Preparation Facility (GZ), The Hong Kong University of Science and Technology (Guangzhou), Guangzhou, Guangdong 511400, China*

*^3^Particles and Catalysis Research Group, School of Chemical Engineering, The University of New South Wales, Sydney, NSW 2052, Australia.*

*^4^Optical Sciences Centre, Swinburne University of Technology, Victoria 3122, Australia*

*^5^Department of Electrical Engineering and Computer Science, University of Arkansas, Fayetteville, AR 72701, United States.*

*^6^RMIT Microscopy and Microanalysis Facility, RMIT University, Melbourne 3000, Australia*

*^7^School of Science, RMIT University, 124 La Trobe Street, Melbourne 3000, Australia*

*^8^Thrust of Advanced Materials, Function Hub, Guangzhou Municipal Key Laboratory of Materials Informatics, The Hong Kong University of Science and Technology (Guangzhou), Guangzhou, Guangdong 511400, China*

*^9^These authors contributed equally*

**Corresponding Authors Email:* [*irfan.haider.abidi@rmit.edu.au*](mailto:irfan.haider.abidi@rmit.edu.au) *and* [*sumeet.walia@rmit.edu.au*](mailto:sumeet.walia@rmit.edu.au%20)


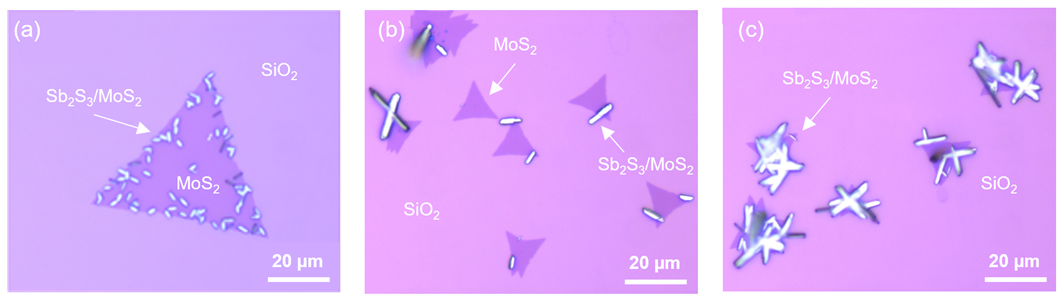
**Figure S1.** (a-c) Optical images revealing MoS_2_ single-crystal offer its surface as a seed layer or template for Sb_2_S_3_ growth in our initial studies, as Sb_2_S_3_ particles are preferentially nucleated at MoS_2_ basal plane, later we optimized the growth conditions to grow 2D sheets of Sb_2_S_3_.


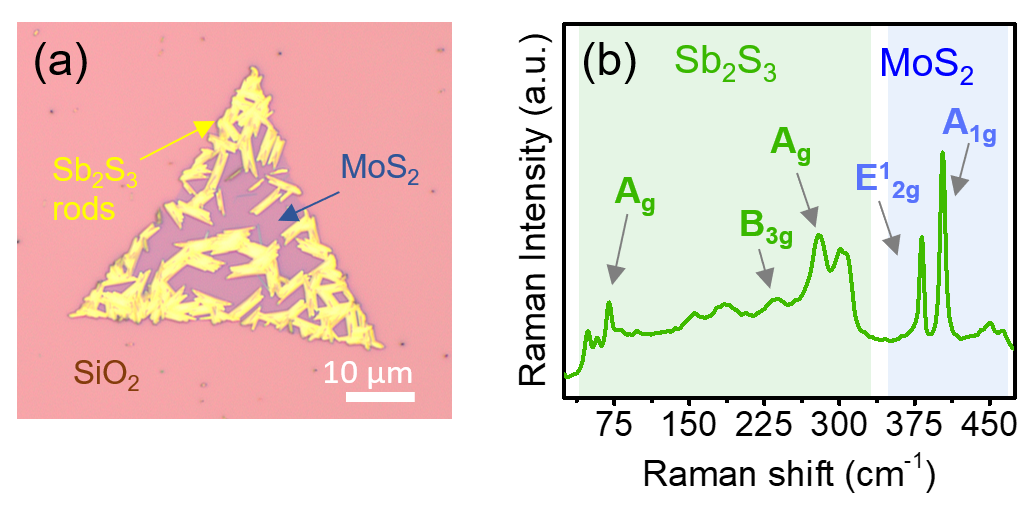


**Figure S2**, a) Optical image of as grown Sb_2_S_3_ on MoS_2_ at 350°C revealing 1D rod-like structures. b) Raman spectrum of 1D Sb_2_S_3_/MoS_2_ flake showing Raman modes corresponding to both Sb_2_S_3_ and MoS_2_.

**
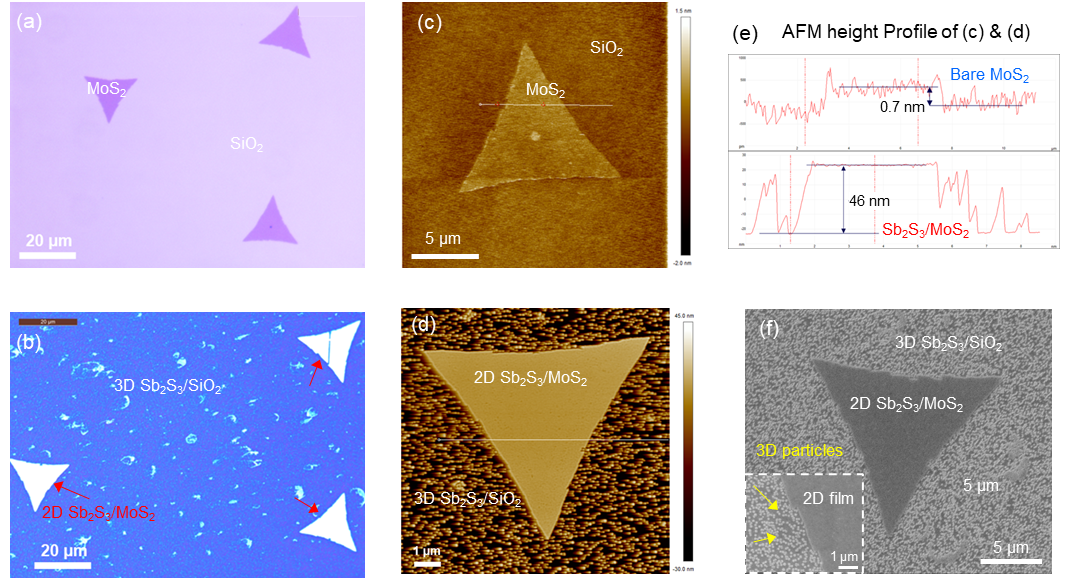
**

**Figure S3.** Optimization of 2D film growth of Sb_2_S_3_ on MoS_2_ flake. Optical and AFM images of (a,c) bare MoS_2_ after 1^st^ CVD growth as template and (b,d) Sb_2_S_3_/ MoS_2_ heterostructure after 2^nd^ growth, respectively. (e) AFM height profile of before and after Sb_2_S_3_ growth on bare MoS_2_, showing increase in thickness of the stack. Notably, smoother surface observed over the MoS_2_ flake suggesting uniform 2D film formation, while rough surface indicating particulates formation is observed away from the MoS_2_ flake. (f) SEM image revealing the 2D Sb_2_S_3_ film growth on MoS_2_, in contrast to 3D particles growth on bare SiO_2_ substrate.


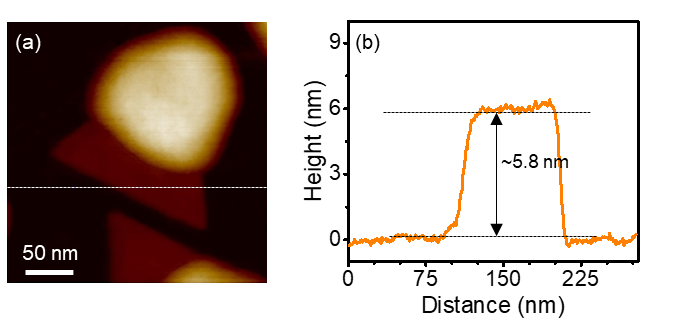


**Figure S4**, (a) AFM image and (b) height profile of the isolated 2D Sb_2_S_3_ flakes achieved through MoS_2_ template-assisted CVD growth.


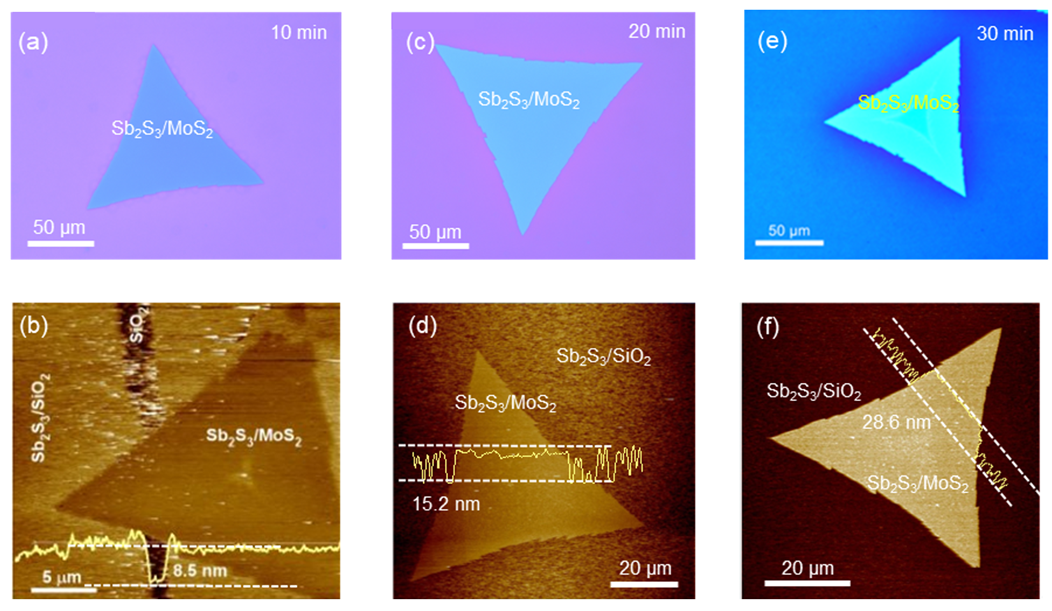
**Figure S5.** Thickness variation of time-dependent growth of Sb_2_S_3_ film on MoS_2_ template. Optical and AFM images of 2D Sb_2_S_3_/MoS_2_ heterostructure after (a,b) 10 minutes, (c,d) 20 minutes and (e,f) 30 minutes of 2^nd^ growth step, respectively. Thickness of the stack increased over the growth time, as revealed by the AFM height profile shown in the inset figures.


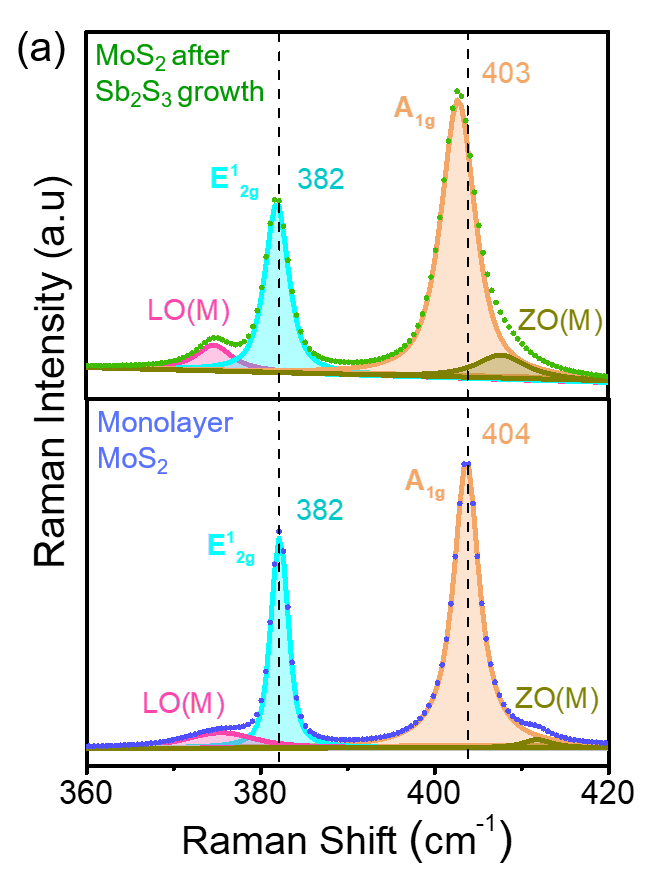


**Figure S6**, Deconvoluted Raman spectra of MoS_2_ peaks before and after Sb_2_S_3_ growth. The red shift in A_1g_ is guided by the dotted line.


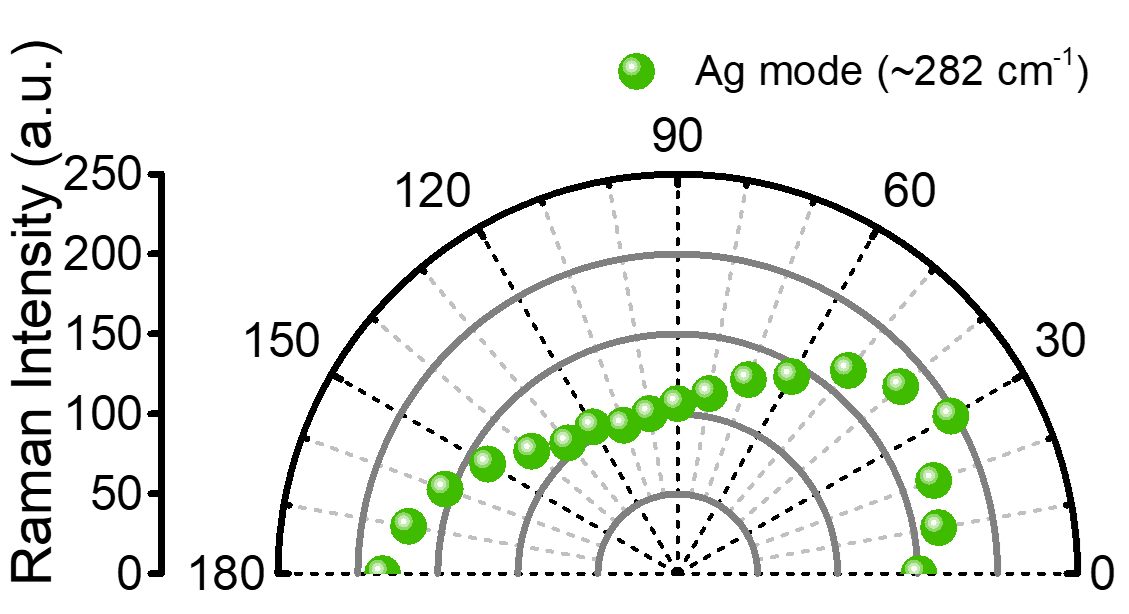


**Figure S7**, Polarized Raman spectroscopy of the Ag mode (~282 cm^-1^) of Sb_2_S_3_ demonstrates angle-dependent Raman intensity shifts.


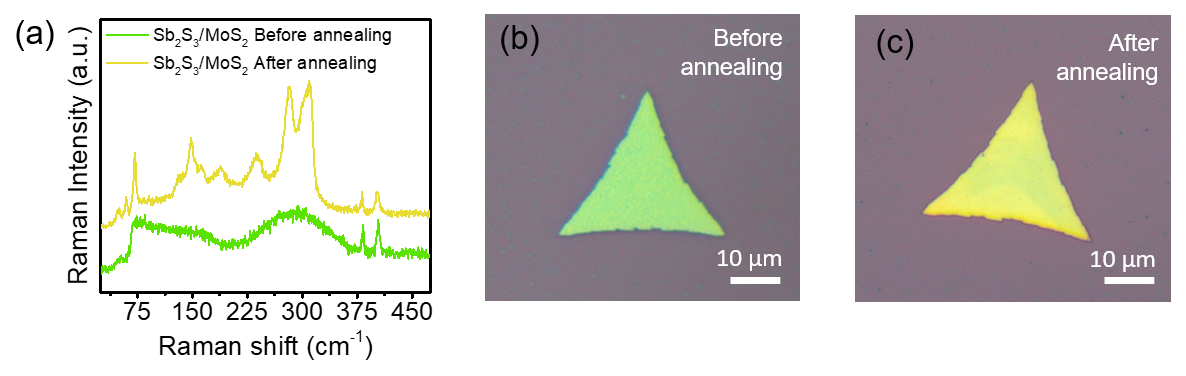


**Figure S8**, (a) Raman spectra (a) and optical images (b), (c) of Sb_2_S_3_/MoS_2_ sample before and after annealing.


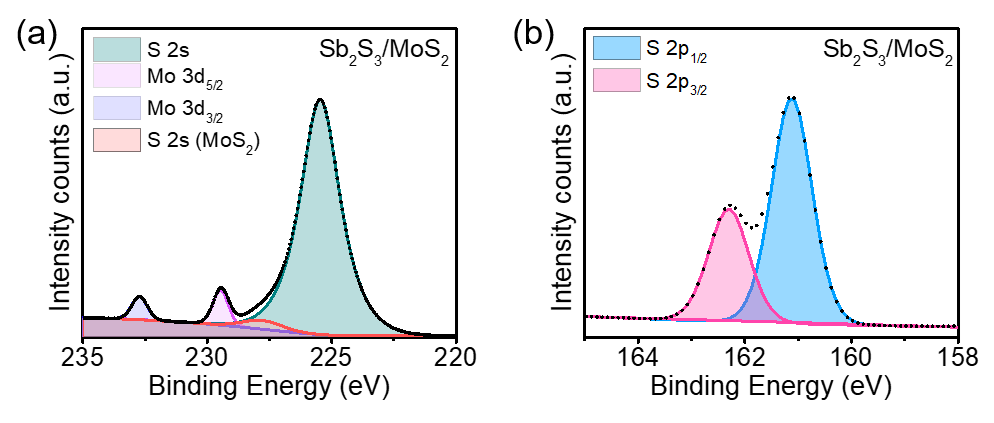


**Figure S9.** XPS spectra of (a) S 2s and (b) S 2p core leveles. Peaks corresponding to Mo 3d from Sb_2_S_3_/MoS_2_ heterostructure shows much broader and quenched peaks compared to the Mo 3d from bare monolayer MoS_2_ shown in main manuscript.


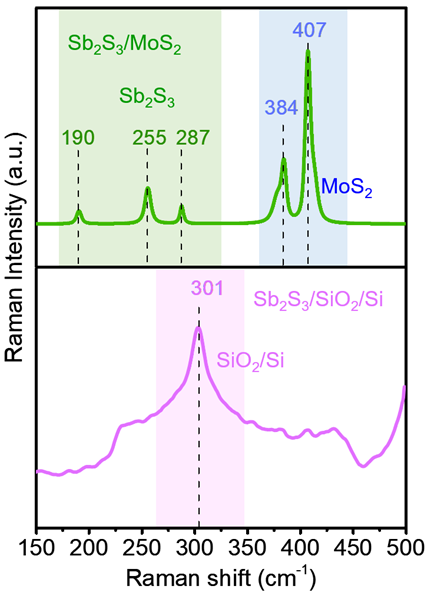


**Figure S10.** Comparison of Raman spectra of Sb_2_S_3_ 3D particles on bare SiO_2_/Si substrate and template-assisted 2D Sb_2_S_3_ grown on MoS_2_ monolayer.


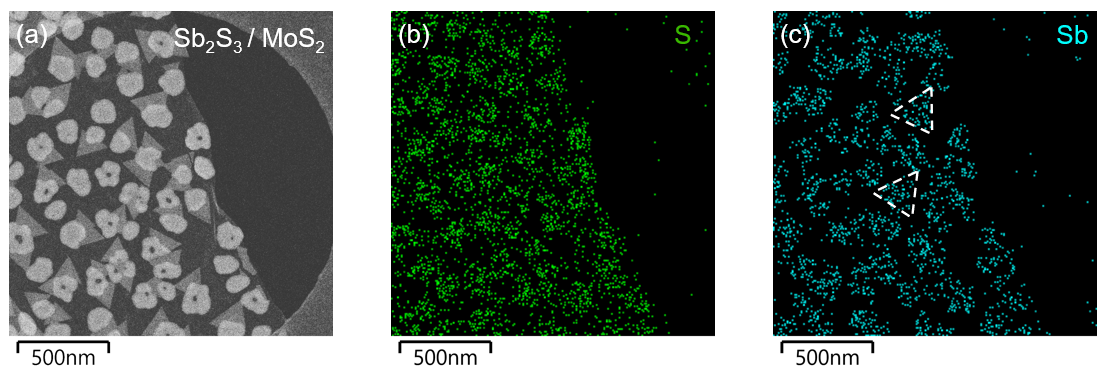


**Figure S11**. (a) STEM image of 2D Sb_2_S_3_ grown on MoS_2_ template, (b) and (c) EDS maps of elemental distribution for S and Sb, respectively.


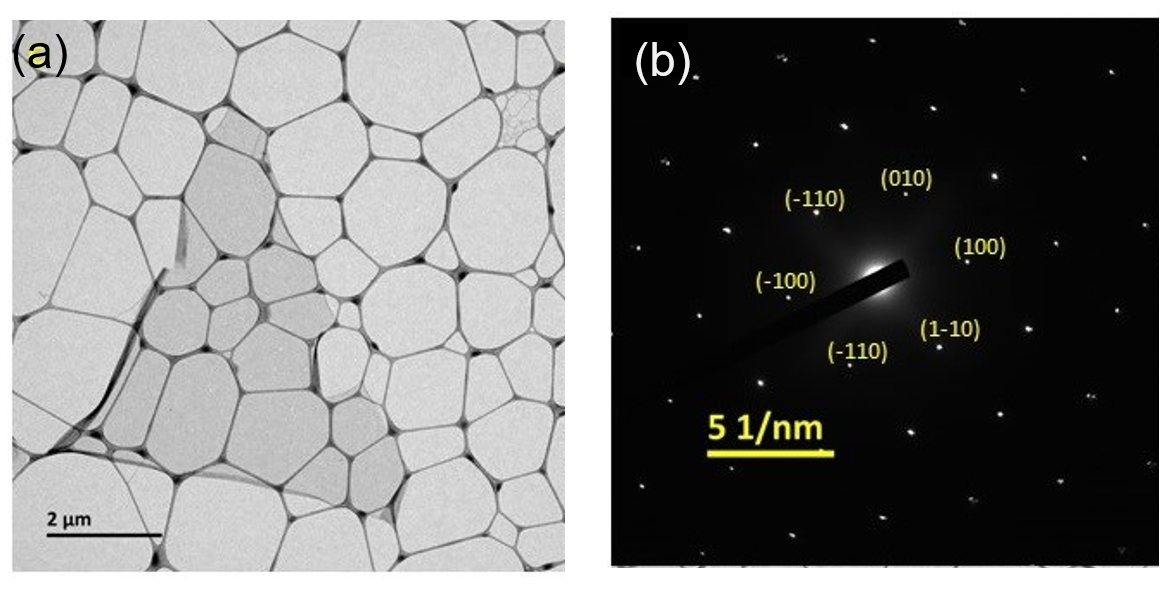


**Figure** **S12**. a) single crystal of MoS_2_ , b) Diffraction pattern from bare MoS_2_ crystal indexed to the 1H phase of MoS_2_


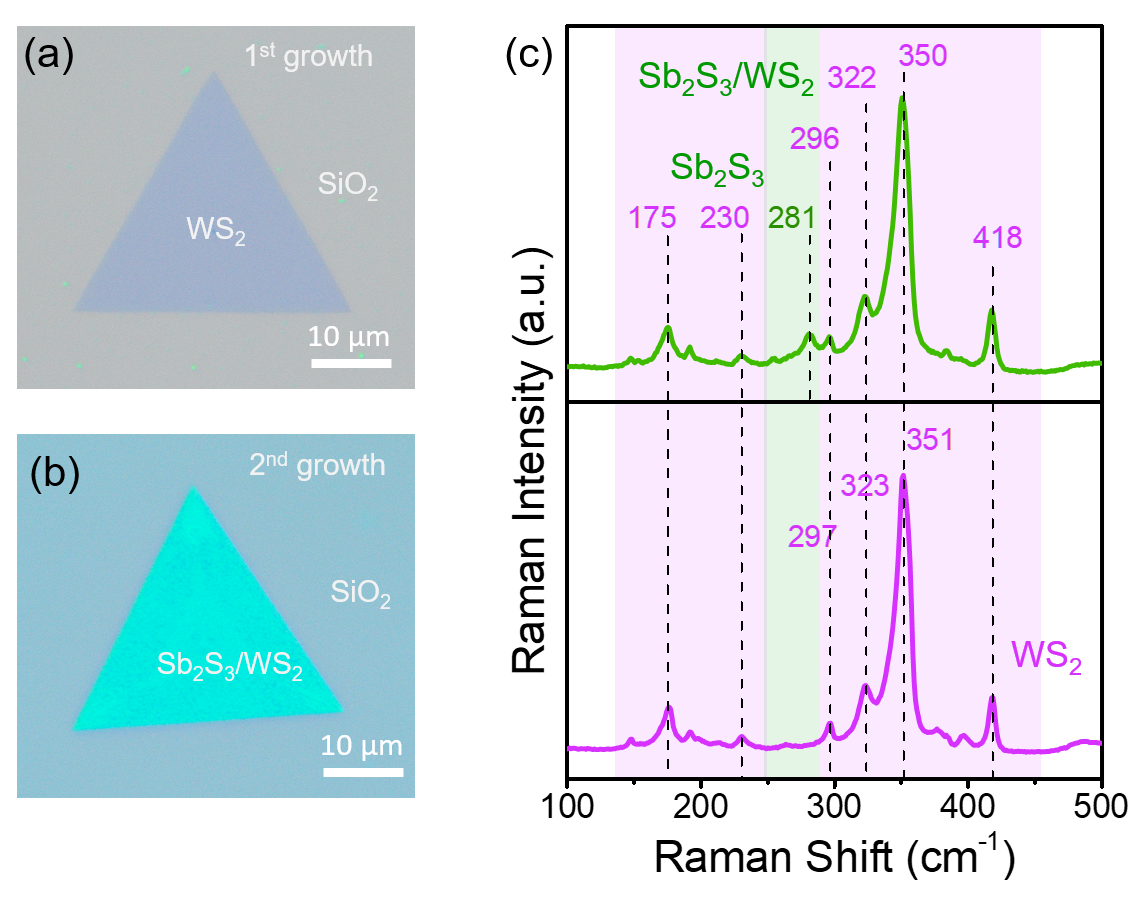


**Figure S13**. Synthesis of Sb_2_S_3_ on other MX_2_ based materials. a) and b) optical image of as grown WS_2_ crystal and Sb_2_S_3_/WS_2_ heterostructure. c) Raman spectra of monolayer WS_2_ template and as grown 2D Sb_2_S_3_ nanosheets grown on WS_2_.


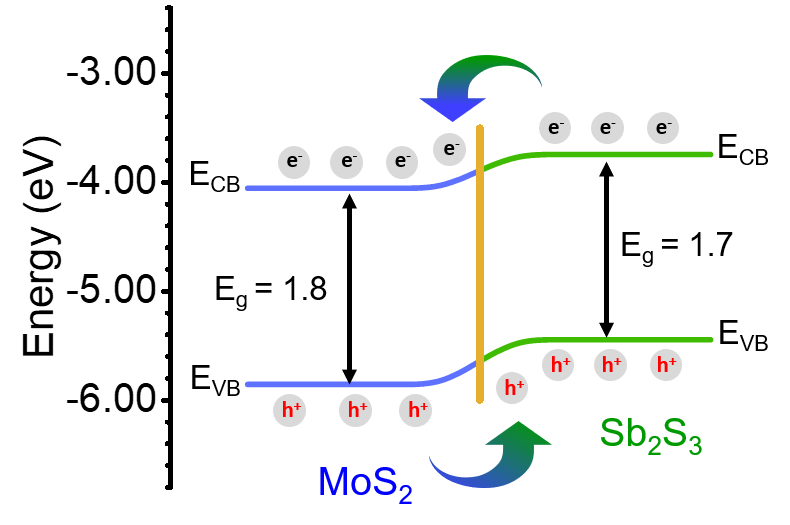


**Figure S14.** Type-II band alignment diagram for the 2D MoS_2_/Sb_2_S_3_ heterostructure. The valence band (VB) and conduction band (CB) energy obtained from literature.^54^.

***Charge carrier concentration (n_e_) calculation***

$n_{e} = c_{i}*\frac{|\left( V_{G} - V_{T} \right)|}{q}$ -------- (1)

where *c*_i_ = 1.15 × 10^−8^ F cm^−2^ is the gate capacitance of the 300 nm SiO_2_ dielectric layer, *V*_G_ and *V*_T_ are gate and threshold voltages, respectively, and *q* is the elementary charge (1.6 × 10^−19^ C). The calculated value of *V*_T_ for monolayer MoS_2_ and Sb_2_S_3_/MoS_2_ heterostructure is -32 V and -57 V respectively (**Figure** S15). The calculated value of n_e_ for monolayer MoS_2_ and Sb_2_S_3_/MoS_2_ at *V*_G_ = -30 V is 1.44×10^11^ and 1.94×10^12^ cm^−2^ respectively.

***Mobility (µ_e_) Calculations***

µ_e_ = $\frac{\Delta I_{DS}}{\Delta V_{G}}\times\frac{L}{W\times c_{i}\times V_{DS}}$ -------- (2)

where µ is the carrier mobility,  $\frac{\Delta I_{DS}}{\Delta V_{G}}$ is derived from the maximum linear slope of the transfer curve in Figure 4e when measured at constant V_DS_ (2V), *c*_i_ is the gate capacitance (1.15 × 10^−8^ F cm^−2^), L and W is channel length and width.

The performance parameters of the fabricated devices are estimated using the following equations:

$\mathrm{Responsivity}\left( R \right)=\frac{I_{ph}}{P_{inc}*A}$ -------- (3)

$Detectivity (D)=R.\sqrt{\frac{A}{2eI_{d}}}$ -------- (4)

where, I_ph_ (τ) is change in photocurrent, P_inc_ is incident optical power density for a specific wavelength, A is the device's active area, and I_d_ is the device's dark current.


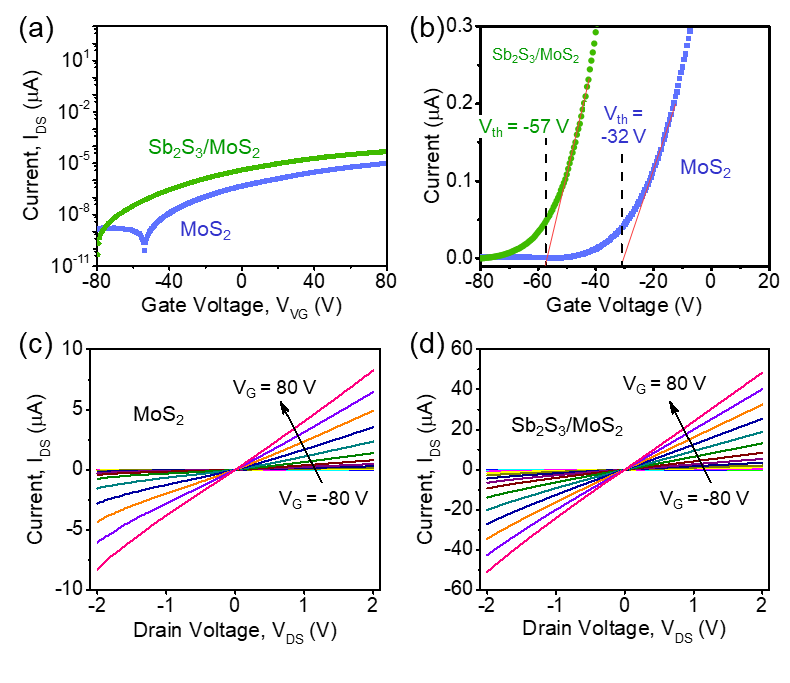


**Figure S15.** (a) Transfer characteristics of FET based on single-crystal MoS_2_ and Sb_2_S_3_/MoS_2_ heterostructure, with current on a log scale. (b) Enlarged view of the linear plot of transfer characteristics of FET, showing the threshold voltage shift between MoS_2_ and Sb_2_S_3_/MoS_2_ heterostructure, suggesting Sb_2_S_3_/MoS_2_ heterostructure relatively n-doped. The output characteristics (*I*_DS_-*V*_DS_) of FETs based on (b) MoS_2_, and (c) MoS_2_/Sb_2_S_3_ heterostructure under applied gate voltage of −80 to +80 V, showing an ohmic behavior.


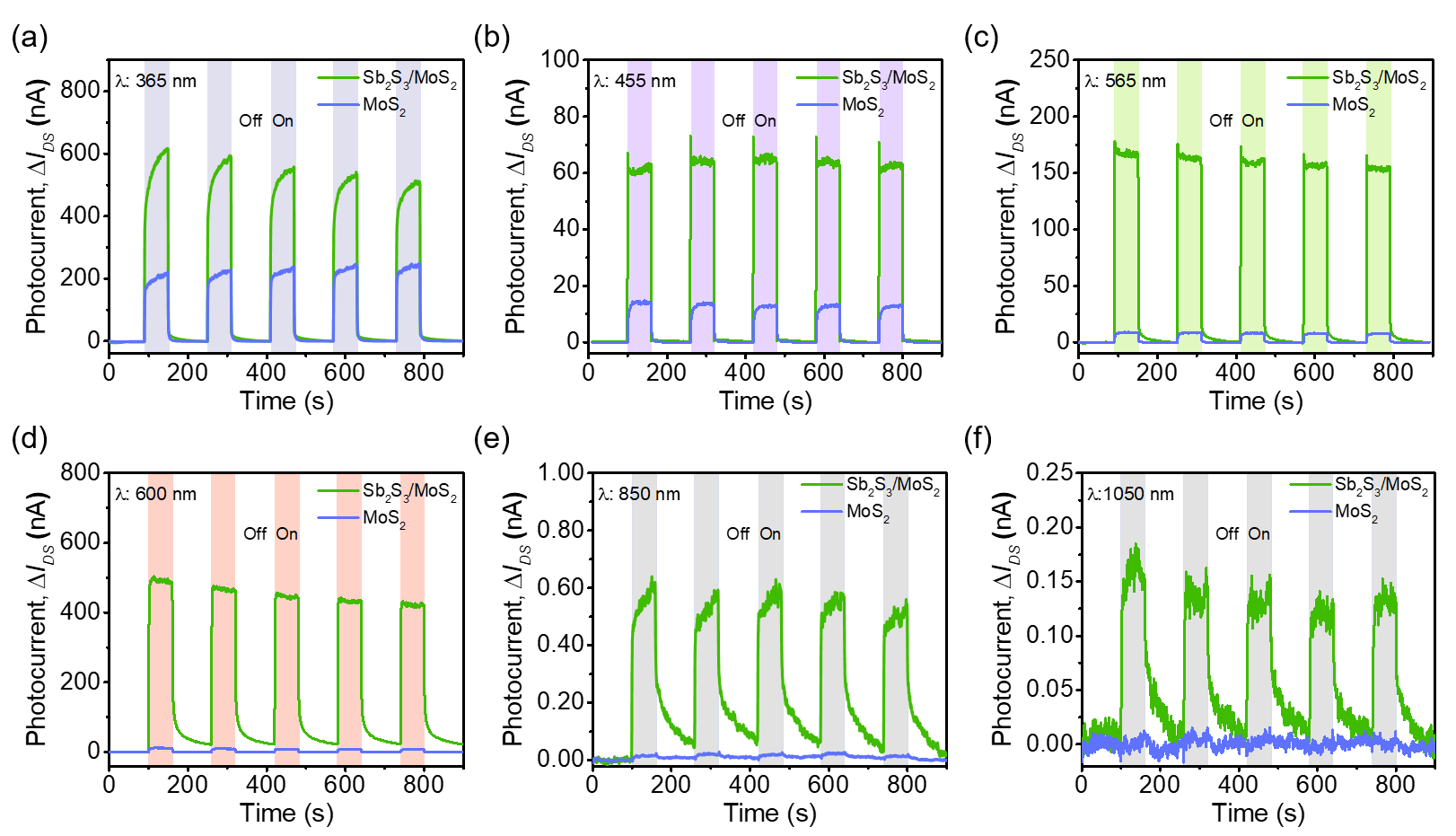
**Figure S16.** Comparison of the photodetector devices based on monolayer MoS_2_ and Sb_2_S_3_/MoS_2_ heterostructure. Transient photo-response measurements under different wavelengths ranging for UV to near IR region at power density of 4 mW/cm^-2^ under constant bias of 2V. a) 365 nm, b) 455 nm, c) 565 nm, d) 660 nm, e) 850 nm and f) 1050 nm.

**Table 1. Comparison table of Sb_2_S_3_ based heterostructure devices.**

| Sb_2_S_3_ based Heterostructure Device | Synthesis process | Thickness achieved | Lateral dimension | Spectral range (nm) | Light source | Light power intensity (mWcm^−2^) | Bias  (V) | Responsivity (A W^−1^) & Detectivity (Jones) | | Ref. |
| --- | --- | --- | --- | --- | --- | --- | --- | --- | --- | --- |
| This work – vdW Template-assisted growth of 2D Sb_2_S_3_ | Chemical vapour deposition | sub-8 nm | ~100 μm (dicated by the base MoS_2_ layer) | 365 – 1050 | 660 nm  and  565 nm | 4 | 2 | 283.4  and  90.5 | 4.7 × 10^12^  and  2.3 × 10^12^ | This work |
| Sb_2_S_3_ nanowire photodetector | Vapour transport | Diameter of 59.6 nm |  | 300 – 800 | 638 nm | 45.2 | 1 | 1152 | 2 × 10^13^ | 1 |
| Sb_2_S_3_ microtube photodetectors | Hydrothermal, solution-based reaction | Diameter of ~ 8 μm | length of 230 μm and width ~10 nm | 350 – 800 | 722 nm | 0.1 | 6 | 82 | 7.6 × 10^10^ | 2 |
| Antimony Chalcogenide van der Waals Nanostructures | Liquid phase exfoliation | 60 nm | N/A | N/A | N/A | N/A | N/A | N/A | N/A | 3 |
| Sb_2_S_3_/MoS_2_ heterostructure as anode | Hydrothermal process | Diameter ~40 nm | >1 μm | N/A | N/A | N/A | N/A | N/A | N/A | 4 |
| Ultrafine MoS_2_/Sb_2_S_3_  Nanorod | wet chemical/ hydrothermal treatment | N/A | N/A | 200 – 1400 | 350 W | N/A | N/A | N/A | N/A | 5 |
| Self-Powered Sb_2_S_3_ Thin-Film Photodetectors | Rapid thermal evaporation | 460 nm | N/A | 300 –750 | 530 nm | 4 | 0 | 1.89 | ~1.7×10^13^ | 6 |
| Ultrathin Sb_2_S_3_ nanosheet anodes | Chemical exfoliation | 2.5 - 3.0 nm | tens of micrometers | 600 – 800 | N/A | N/A | N/A | N/A | N/A | 7 |
| van der Waals (vdW) 2D/1D PbI_2_/Sb_2_S_3_  heterojunction photodetector | Hydrothermal, solution-based reaction; dry transfer | N/A | microrods exhibit lengths distributed at >110 μm | 400 – 750 | 455 nm  and  730 nm | 0.01  and  0.02 | -3 | 156.3  /120.4 | 3.16×10^13^  and 2.45×10^12^ | 8 |
| Sb_2_Te_3_ /MoS_2_ heterojunction photodetectors | Mechanical exfoliation | Sb_2_Te_3_ (~65 nm) | tens of micrometers | 500 – 900 | 660 nm | N/A | 0‑1 | 0.17/  ~0.51 | N/A | 9 |

**References:**

(1) Zhong, M.; Wang, X.; Liu, S.; Li, B.; Huang, L.; Cui, Y.; Li, J.; Wei, Z. High-performance photodetectors based on Sb2S3 nanowires: Wavelength dependence and wide temperature range utilization. *Nanoscale* **2017**, *9* (34), 12364-12371. DOI: 10.1039/c7nr03574h.

(2) Fu, S.; Liu, X.; Dou, H.; Ali, R.; Zeng, A.; Man, J.; Zheng, X.; Wang, H. E. Single-crystalline Sb2S3 microtubes for high-performance broadband visible photodetection. *Journal of materials chemistry. A, Materials for energy and sustainability* **2024**, *12* (41), 28012-28022. DOI: 10.1039/d4ta04573d.

(3) Gusmão, R.; Sofer, Z.; Luxa, J.; Pumera, M. Antimony Chalcogenide van der Waals Nanostructures for Energy Conversion and Storage. *ACS Sustainable Chemistry & Engineering* **2019**, *7* (18), 15790-15798. DOI: 10.1021/acssuschemeng.9b04415.

(4) Zhang, Z.; Zhao, J.; Xu, M.; Wang, H.; Gong, Y.; Xu, J. Facile synthesis of Sb2S3/MoS2 heterostructure as anode material for sodium-ion batteries. *Nanotechnology* **2018**, *29* (33), 335401. DOI: 10.1088/1361-6528/aac645.

(5) Li, W.; Ma, T. H.; Dang, Y. Y.; Liu, X. Y.; Li, J. Y.; Wang, C. Y. Ultrafine MoS2/Sb2S3 Nanorod Type‐II Heterojunction for Hydrogen Production under Simulated Sunlight. *Advanced materials interfaces* **2022**, *9* (15), n/a. DOI: 10.1002/admi.202200119.

(6) Lin, X.; Deng, H.; Jia, Y.; Wu, Z.; Xia, Y.; Wang, X.; Chen, S.; Cheng, Y.; Zheng, Q.; Lai, Y.; et al. Self-Powered Sb2S3 Thin-Film Photodetectors with High Detectivity for Weak Light Signal Detection. *ACS applied materials & interfaces* **2022**, *14* (10), 12385-12394. DOI: 10.1021/acsami.1c25256.

(7) Yao, S.; Cui, J.; Deng, Y.; Chong, W. G.; Wu, J.; Ihsan-Ul-Haq, M.; Mai, Y.-W.; Kim, J.-K. Ultrathin Sb2S3 nanosheet anodes for exceptional pseudocapacitive contribution to multi-battery charge storage. *Energy Storage Materials* **2019**, *20*, 36-45. DOI: <https://doi.org/10.1016/j.ensm.2018.11.005>.

(8) Fu, S.; Liu, X.; Man, J.; Ou, Q.; Zheng, X.; Liu, Z.; Zhu, T.; Wang, H. E. 2D/1D PbI2/Sb2S3 van der Waals heterojunction for highly sensitive and broadband photodetectors. *Journal of materials chemistry. C, Materials for optical and electronic devices* **2024**, *12* (9), 3353-3364. DOI: 10.1039/d3tc04279k.

(9) Wang, H.; Gui, Y.; Dong, C.; Altaleb, S.; Nouri, B. M.; Thomaschewski, M.; Dalir, H.; Sorger, V. J. Self-powered broadband photodetector based on MoS2/Sb2Te3 heterojunctions: a promising approach for highly sensitive detection. *Nanophotonics (Berlin, Germany)* **2022**, *11* (22), 5113-5119. DOI: 10.1515/nanoph-2022-0413.
